# Supplementary material for: ThermiQuant™ AquaStream: A portable instrument for quantitative colorimetric isothermal nucleic acid amplification reactions in paper and tube formats
Source: PLoS One. 2026 May 15;21(5):e0348607. doi: 10.1371/journal.pone.0348607 (PMC13178971; doi:10.1371/journal.pone.0348607)
Supplement: S1 File — All supporting figures and tables are included. (PDF) [file pone.0348607.s001.pdf]

Supporting Information

for

ThermiQuant™ AquaStream: A portable instrument for quantitative colorimetric isothermal nucleic acid amplification reactions in paper and tube formats

Bibek Raut[1], Gopal Palla[2], Jiangshan Wang[2], Skyler Campbell[3], Virendra Kumar[2], Kyungyeon Ra[2], Mohit S. Verma[1,2,4,5,6*]

[1]Weldon School of Biomedical Engineering, Purdue University, West Lafayette, IN, USA

[2]Department of Agricultural and Biological Engineering, Purdue University, West Lafayette, IN, USA

[3]Department of Biomedical Engineering, Duke University, Durham, NC, USA

[4]Purdue Institute of Inflammation, Immunology and Infectious Disease, Purdue University, West Lafayette, IN 47906, USA

[5]Purdue Institute for a Sustainable Future, Purdue University, West Lafayette, IN, USA

[6]Bindley Bioscience Center, Purdue University, West Lafayette, IN, USA

*Corresponding author

Email: msverma@purdue.edu (MV)

Contents

1. Supporting Figures 3
   S1 Fig. Step-by-step visual guide to assemble ThermiQuant™ AquaStream for the first time 3
   S2 Fig. 3D view of device and cartridge assembly. 5
   S3 Fig. ThermiQuant™ AquaStream user manual. 6
   S4 Fig. µPAD cartridge housing assembly 7
   S5 Fig. Timelapse of NTC-only reactions in tubes 7
   S6 Fig. Determination of positivity threshold, LOD, and LOQ in liquid colorimetric LAMP 8
   S7 Fig. Determination of positivity threshold, LOD, and LOQ in paper colorimetric LAMP 9
   S8 Fig. Effect of DNA concentration on the initial pH and hue value 10
   S9 Fig. Inter-run variation in Tq of colorimetric paper LAMP assays 11
2. Supporting Tables 12
   S1 Table. BOM of ThermiQuant™ MegaScan 12
   S2 Table. Orf7ab synthetic DNA sequence 13
   S3 Table. Digital PCR (dPCR) primers and probes 13
   S4 Table. LAMP primers 13

References 14

1. Supporting Figures

ThermiQuant™ AquaStream Assembly Guide

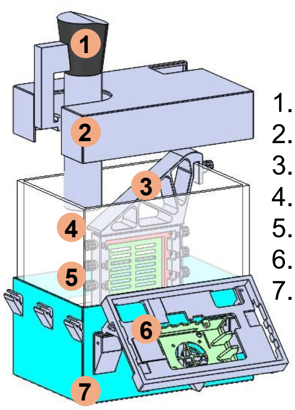

Assembly Parts

1. Water heater rod
2. Tank cover
3. Cartridge holder
4. Glass water tank
5. Backlight
6. Camera & monitor
7. Tank box

Step 1: Place backlight at the back of tank-box such that the USB-C charging port is facing up

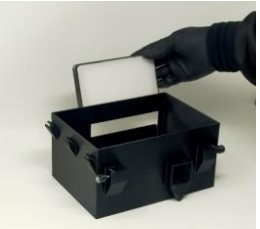

Step 2: Place the glass tank inside tank-box making sure it is touching the base of the tank-box

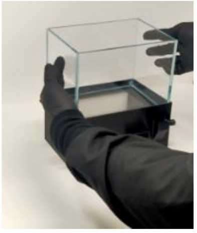

Step 3: Insert red-camera into the camera slot in the tank-box. The camera ribbon should face down

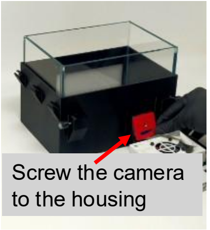

Step 4: Place the monitor in the tank-box holder & secure its leg supports with screw-locks

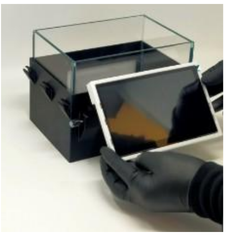

Step 5: Connect USB-C cables to both monitor and backlight and fix the cable on the side wall open ring structure

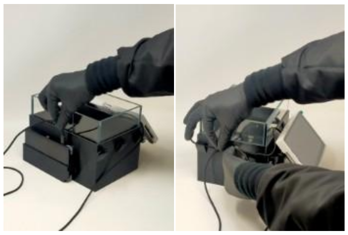

Step 6: Place the water heater rod at the center of the left side wall & screw it softly to the tank wall

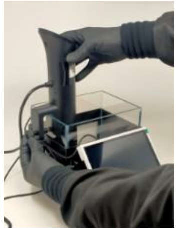

Step 7: Remove tank cover & pour about 2.5 liters (~0.7 gallon) of water with surfactant (5-10 mL Tween-20)

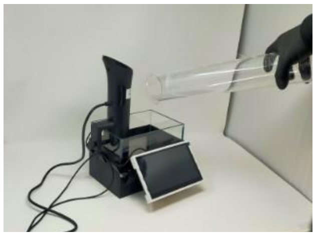

Step 8: Place tank cover. You may adjust the placement of heater rod to fit the cover

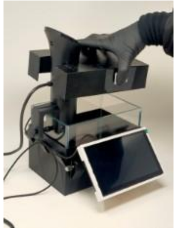

S1 Fig. Step-by-step visual guide to assemble ThermiQuant™ AquaStream for the first time.

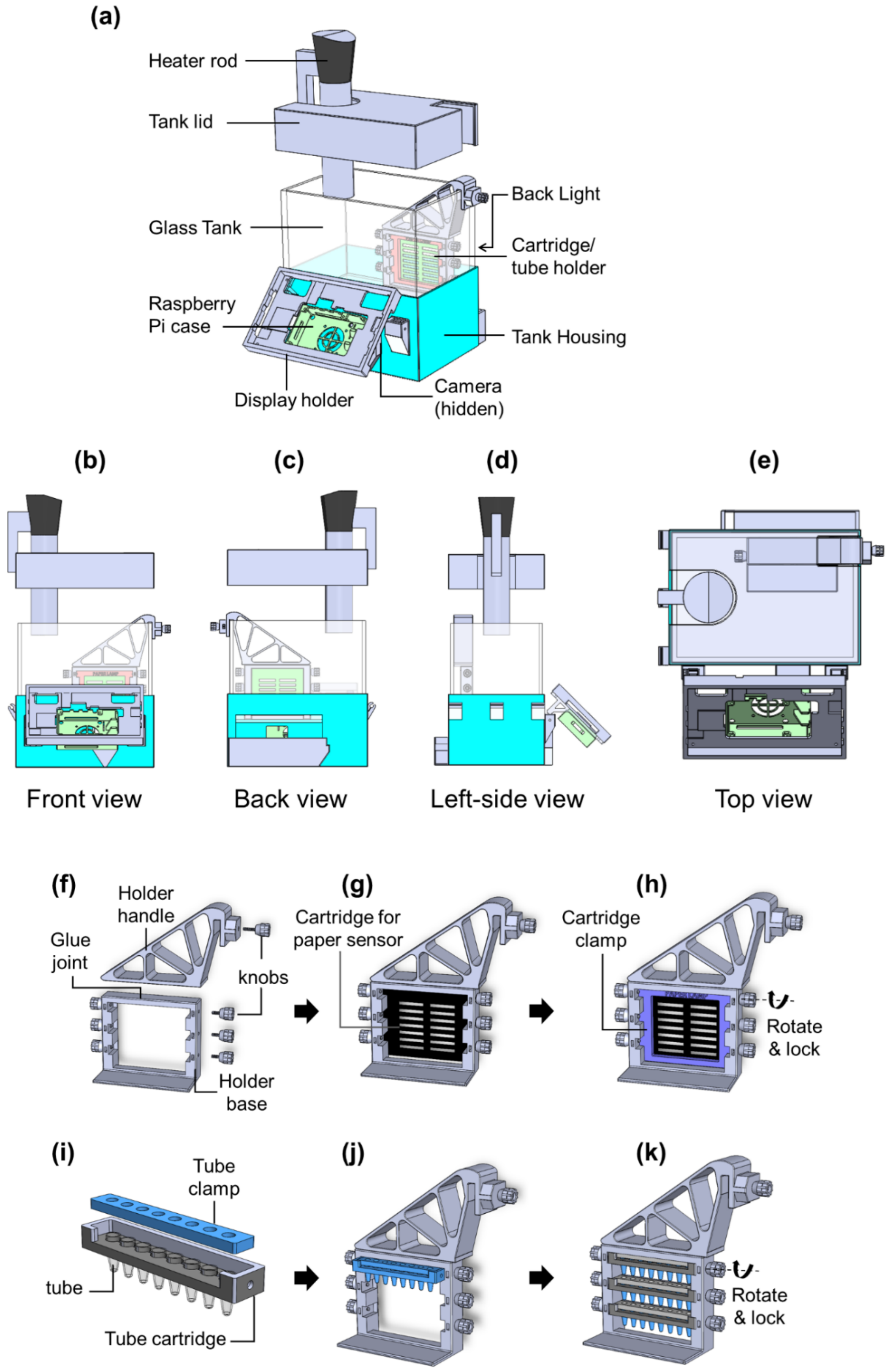
(a)
Heater rod
Tank lid
Glass Tank
Back Light
Cartridge/
tube holder
Raspberry
Pi case
Tank Housing
Display holder
Camera
(hidden)
(b)
(c)
(d)
(e)
Front view
Back view
Left-side view
Top view
(f)
Holder
handle
Glue
joint
knobs
Holder
base
(g)
Cartridge for
paper sensor
(h)
Cartridge
clamp
Rotate
& lock
(i)
Tube
clamp
tube
Tube cartridge
(j)
(k)
Rotate
& lock

S2 Fig. 3D view of device and cartridge assembly. (a) Exploded view of the instrument. (b) Front view. (c) Back view. (d) Left-side view. (e) Top view. (f) Exploded assembly view of the cartridge/tube holder showing two parts, the holder handle and the holder base, which need to be glued together using superglue. The knobs are fitted with M3 screws and nuts to secure the holder to the tank wall or to lock the cartridges in place. (g) To load the paper sensor cartridge, first place the cartridge, then (h) position the cartridge clamp and lock it with the knobs by rotating clockwise for the right side and counterclockwise for the left side. (i) Assembled view of the tube-inside-tube cartridge. (j) Insert the assembled tube cartridge into the cartridge holder, and (k) lock it in place before placing it inside the water bath.

ThermiQuant™ AquaStream User Manual

Step 1: If not filled already, remove tank cover & pour about 2.5 liters (~0.7 gallon) of water with surfactant (10 mL Tween-20).

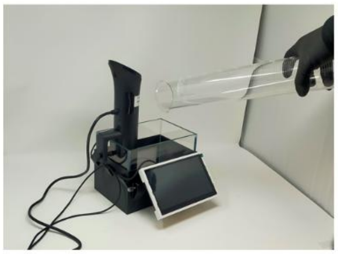

Step 2: Cover the tank & power the heater (set at 149 °F /65°C). Also, power the monitor & backlight. It takes ~10 min to heat water.

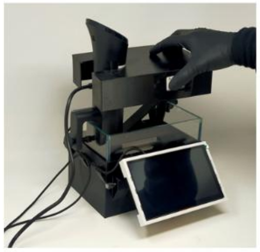

Step 3A: After water reaches 65°C, prepare to load the cartridge inside the holder by first inserting cartridge then adding a rectangular support.

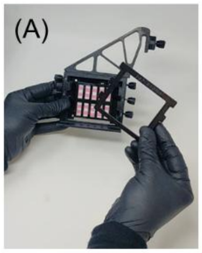

Step 4B: (B) Lock the cartridge & support using screw locks by rotating in opposite directions.

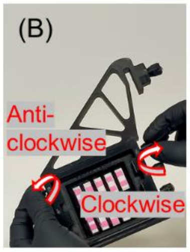

Step 5A: Gently insert the cartridge holder and make sure it is touching the back wall and lays flat on the tank.

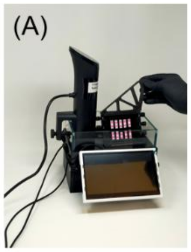

Step 5B: Then gently secure the cartridge to right side wall using screw lock. Do not tighten too much.

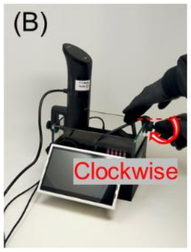

Step 6. Cover the tank with lid to eliminate outside light and reduce water evaporation.

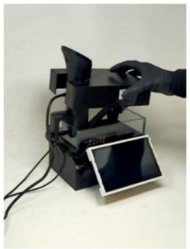

Step 7. In the software select the reaction type and press start to begin the reaction.

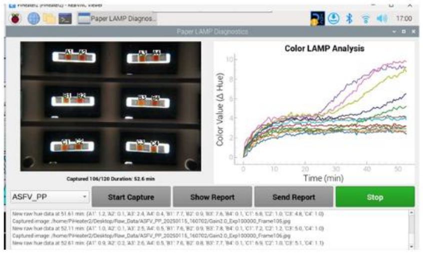

Step 8. After 60 minutes, a result summary screen appears that shows the reaction results with quantitative information.

Label	Result	Tq (min)
A1	Positive	35
A2	Negative	--
B1	Positive	30
B3	Negative	--

S3 Fig. ThermiQuant™ AquaStream user manual.

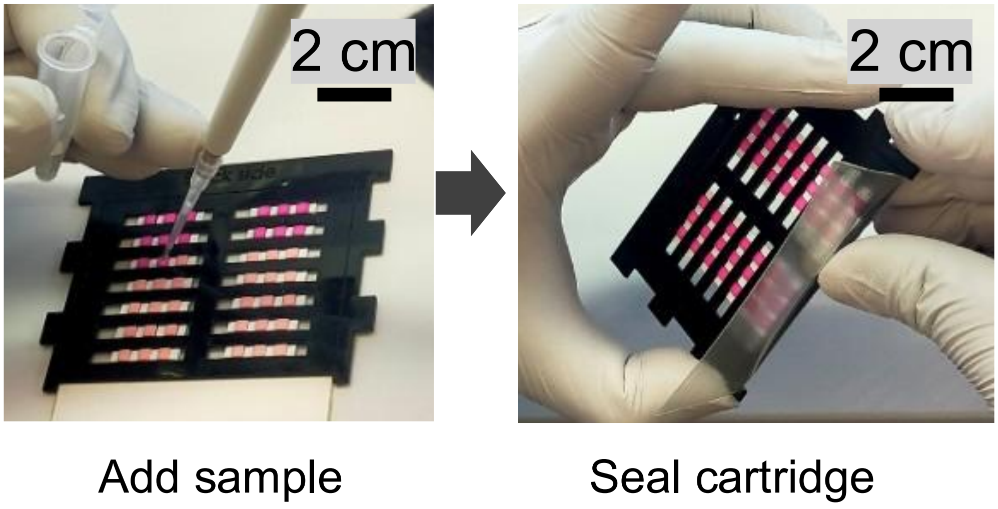

S4 Fig. µPAD cartridge housing assembly. Sample pipetting into µPADs pre-dried with reagents required for LAMP amplification, followed by sealing with PCR tape. Each µPAD zone was loaded with 7.5 µL of sample by pipette, after which the strip was immediately sealed with transparent PCR tape to prevent evaporation and cross-contamination during incubation.

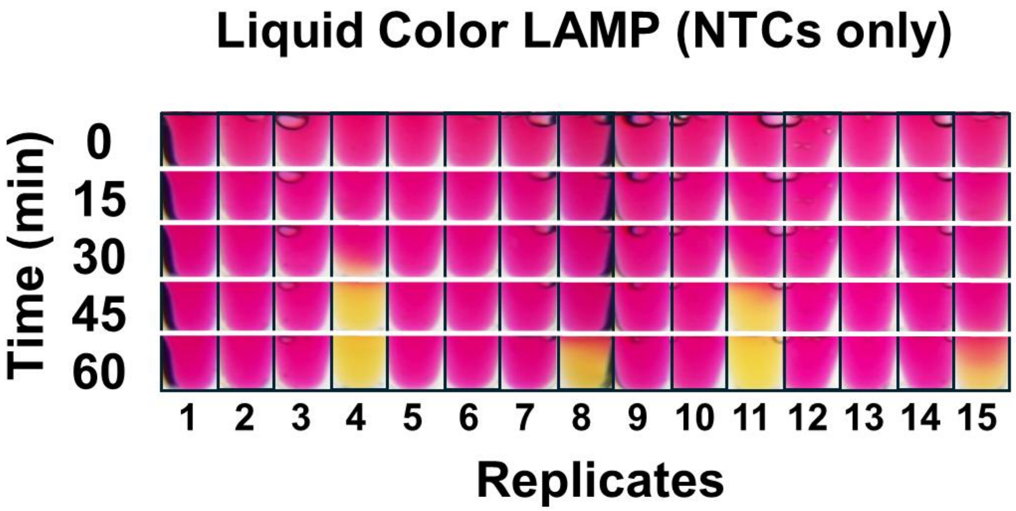

S5 Fig. Timelapse of NTC-only reactions in tubes. Liquid Color LAMP was used to test 15 additional NTC replicates to determine positivity threshold.

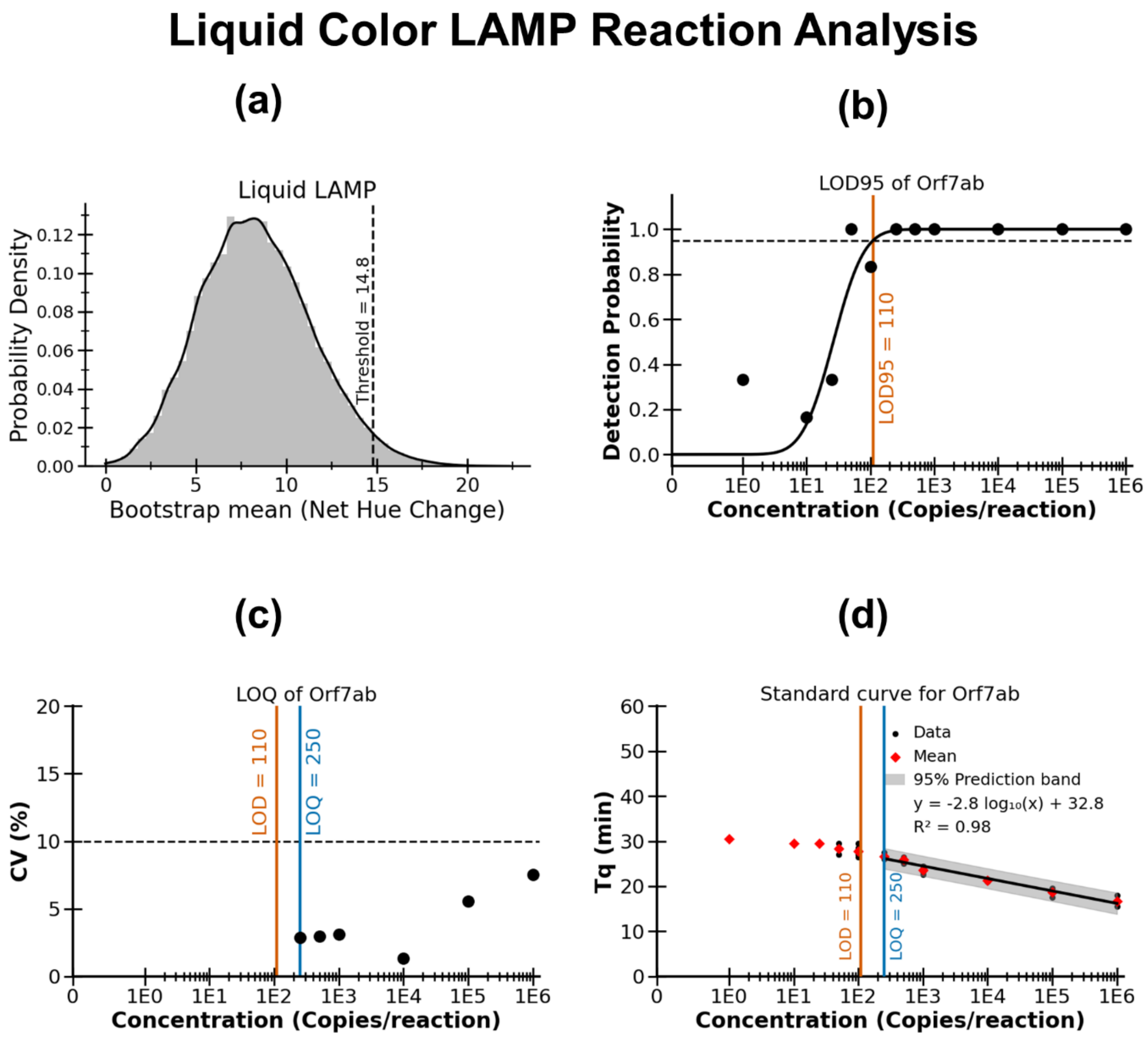

S6 Fig. Determination of positivity threshold, LOD, and LOQ in liquid colorimetric LAMP. (a) Probability density plot of the mean bootstrap net hue change derived from a total of n = 21 NTCs, used to determine the positivity threshold as the 95% upper confidence limit of the bootstrap mean. (b) Probit regression used to determine the LOD95 (95% detection probability). (c) LOQ defined as the lowest analytical concentration tested with a coefficient of variation (CV) ≤ 10% of the quantification time (Tq). (d) Standard curve generated using concentrations above the LOQ, also showing Tq mean and raw values for all positive samples, including those falling below the LOD95.

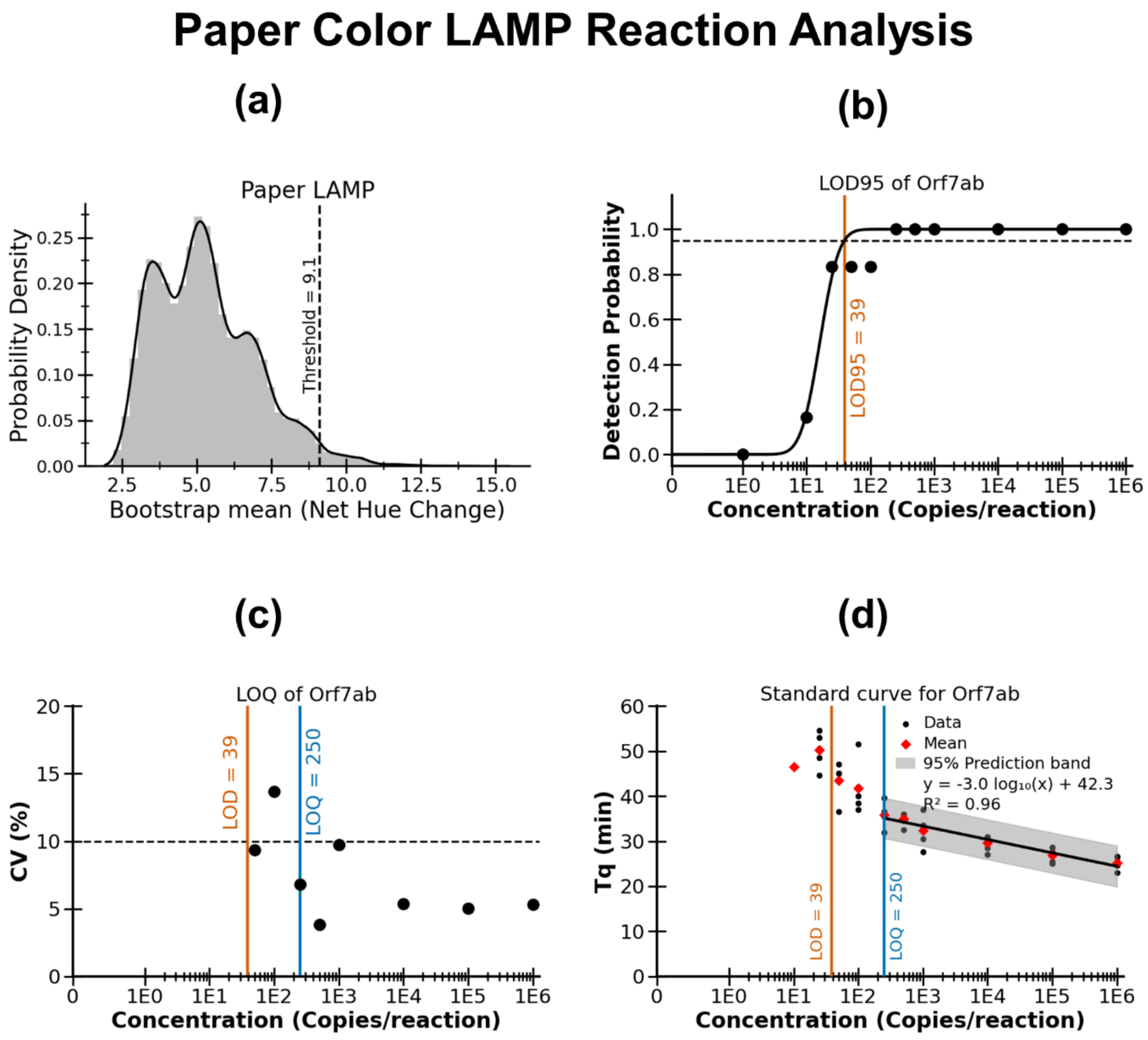

S7 Fig. Determination of positivity threshold, LOD, and LOQ in paper colorimetric LAMP. (a) Probability density plot of the mean bootstrap net hue change derived from a total of n = 14 NTCs, used to determine the positivity threshold as the 95% upper confidence limit of the bootstrap mean. (b) Probit regression used to determine the LOD95 (95% detection probability). (c) LOQ defined as the lowest analytical concentration tested with a coefficient of variation (CV) ≤ 10% of the quantification time (Tq). (d) Standard curve generated using concentrations above the LOQ, also showing Tq mean and raw values for all positive samples, including those falling below the LOD95.

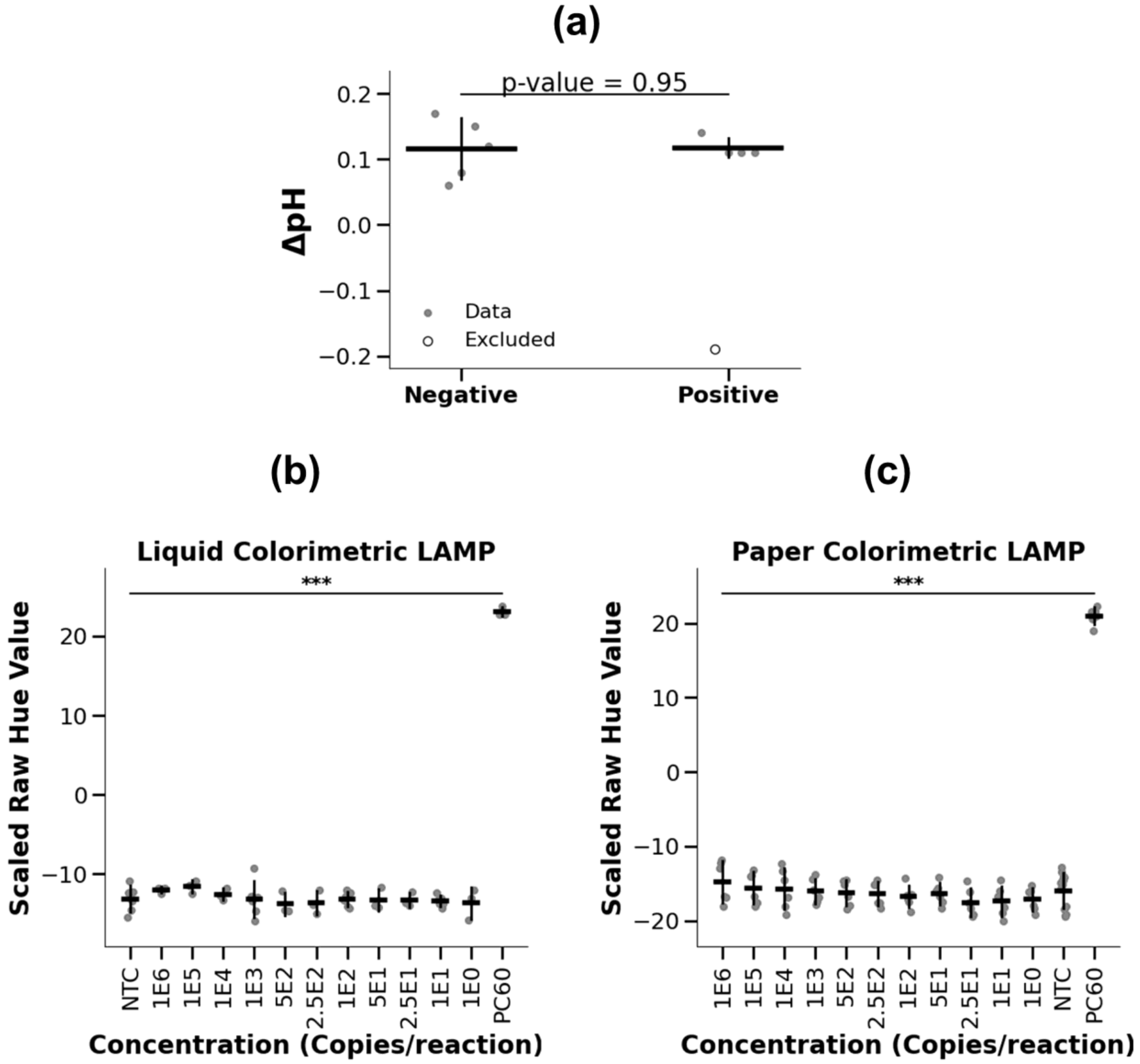

S8 Fig. Effect of DNA concentration on the initial pH and hue value. (A) Change in pH (ΔpH) between the first and second pH measurements for negative (water-only) and positive (DNA-spiked, 40,000 copies/µL) samples. Error bars represent mean ± SD. One positive replicate was excluded as indicated. No significant difference was observed (Welch's t-test, $p = 0.95$). (B) Raw hue values of liquid colorimetric LAMP reactions measured at the initial time point (0 min) across different DNA concentrations (copies per reaction). (C) Raw hue values of paper-based colorimetric LAMP reactions measured at 0 min across the same DNA concentrations. Error bars represent the mean ± SD. NTC denotes the no-template control measured at 0 min. PC60 denotes the positive control (1E6 copies per reaction) measured at 60 min after amplification. No significant differences in hue values were observed between the NTC at 0 min and any DNA concentration measured at 0 min. In contrast, a significant difference ($p < 0.001$) was observed between the NTC (0 min) and PC60.

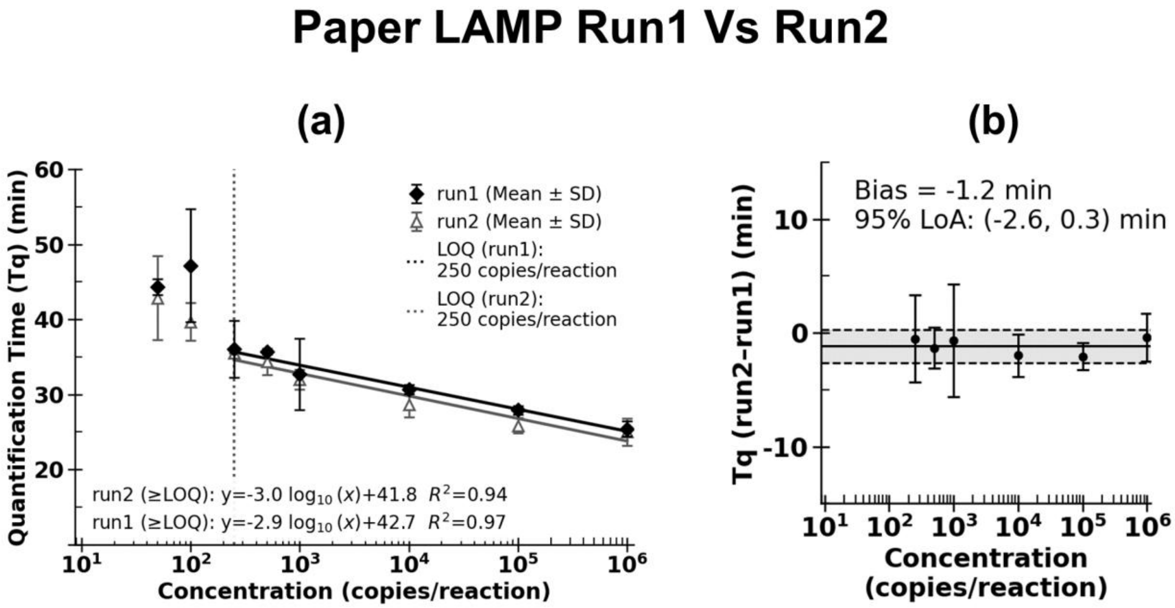

S9 Fig. Inter-run variation in Tq of colorimetric paper LAMP assays. (A) Quantification time (Tq) plotted against input DNA concentration (log scale) for µPAD assays in run 1 and run 2 at and above the LOD95, with linear regression performed only for concentrations at or above the LOQ. (B) Bland–Altman analysis comparing the differences in Tq between runs.

2. Supporting Tables

S1 Table. BOM of ThermiQuant™ MegaScan. All reported prices are for Oct 2025.

Bill of materials for the ThermiQuant™ AquaStream

S.N.	Parts Name	Manufacturer	Vendor	Vendor Parts Number	Units	Price/ Unit (USD)	Total Price (USD)
**1**	Raspberry Pi 4B (4GB)	Raspberry Pi Ltd	Digikey, USA	2648-SC0194(9)-ND	1	55	55
**2**	microSD card (64 GB)	Amazon	Amazon, USA	ASIN: B08TJTB8XS	1	8	7.54
**3**	Power Supply Adapter (5V, 3A)	LitStar	Amazon, USA	ASIN: B08523QCT6	2	10	19.98
**4**	Arducam 16 Megapixel camera	Arducam	Arducam, USA	IMX519	1	30	29.99
**5**	Anova Nano 3.0 Sous Vide Precision Cooker	Anova	Amazon, USA	ASIN: B0BQ93XGWC	1	72	72.35
**6**	Small Nano Rimless Tank (1.1 gallon)	awxzom tank	Amazon, USA	ASIN: B0D3F36CTY	1	29.99	29.99
**7**	Touchscreen Monitor (7 inches, 800x480 pixel)	Freenove	Amazon, USA	ASIN: B0B44VZTRG	1	49.95	49.95
**8**	Rechargeable White LED Light (4000mAh)	Weilisi	Amazon, USA	ASIN: B09C5XKW64	1	29.99	29.99
**9**	3D Printer Filament (PETG and PC, 1 kg)	OVERTURE	Amazon, USA	ASIN: B0BQR8VGSR (PC White); B07PGYHYV8 (PETG Black)	1	22.99	22.99
**10**	M3 Hex socket head screw, spacer & nuts kit (stainless steel)	WZHUIDA	Amazon, USA	ASIN: B0CNJW37NJ	1	8.99	8.99
						**Total**	**326.77**

Synthetic DNA, LAMP Primers, and qPCR probes and primers

We used dPCR for absolute quantification, and LAMP was used for testing both µPAD and liquid-tube based assay. This study builds on a previously reported design of µPAD-based LAMP reactions targeting the *orf7ab* region of SARS-CoV-2[1–3].

S2 Table. Orf7ab synthetic DNA sequence.

Sequence (5'-3') target sequence of *orf7ab*
ATGAAAATTATTCTTTTCTTGGCACTGATAACACTCGCTACTTGTGAGCTTTATCACT ACCAAGAGTGTGTTAGAGGTACAACAGTACTTTTAAAAGAACCTT GCTCTTCTGGAACATACGAGGGCAATTCACCATTTCATCCTCTAGCTGATAACAAAT TTGCACTGACTTGCTTTAGCACTCAATTTGCTTTTGCTTGTCCTGAC GGCGTAAAACACGTCTATCAGTTACGTGCCAGATCAGTTTCACCTAAACTGTTCATC AGACAAGAGGAAGTTCAAGAACTTTACTCTCCAATTTTTCTTATTGT TGCGGCAATAGTGTTTATAACACTTTGCTTCACACTCAAAAGAAAGACAGAATGAT TGAACTTTCATTAATTGACTTCTATTTGTGCTTTTTAGCCTTTCTGCTATTCCTTGTTT TAATTATGCTTATTATCTTTTGGTTCTCACTTGAACTGCAAGATCATAATGAAACTTG TCACGCCTAA

S3 Table. Digital PCR (dPCR) primers and probes. Same as previous report[3].

dPCR Primers	Sequence (5'-3')	Vendor
orf7ab_PCR2_FWD	GAGGGCAATTCACCATTTCATC	Life Science Tech
orf7ab_PCR2_REV	AAACTGATCTGGCACGTAACT	Life Science Tech
Probe	/56-FAM/TT TGC TTG T/ZEN/C CTG ACG GCG TAA A/3IABkFQ/	IDT

S4 Table. LAMP primers. Same as previous report[3]

Primers	Sequence (5'-3')	Vendor
SC2.orf7ab.1_F3	CGGCGTAAAACACGTCTA	IDT
SC2.orf7ab.1_B3	GCTAAAAAGCACAAATAGAAG TC	IDT
SC2.orf7ab.1_FIP	GGAGAGTAAAGTTCTTGAACTT CCTAGTTACGTGCCAGATCAG	IDT
SC2.orf7ab.1_BIP	TGCGGCAATAGTGTTTATAAC ACTATGAAAGTTCAATCATTCT GTCT	IDT
SC2.orf7ab.1_LF	TGTCTGATGAACAGTTTAGGT GAAA	IDT
SC2.orf7ab.1_LB	TTGCTTCACACTCAAAAGAA	IDT

References

1. Davidson, J. L. *et al.* A paper-based colorimetric molecular test for SARS-CoV-2 in saliva. *Biosensors and Bioelectronics: X* **9**, 100076 (2021).
2. Wang, J. *et al.* Fabrication of a paper-based colorimetric molecular test for SARS-CoV-2. *MethodsX* **8**, 101586 (2021).
3. Raut, B. *et al.* ThermiQuant(TM) MegaScan: High-throughput isothermal reactor with quantitative colorimetric readout for paper-based nucleic acid amplification tests. 2026.01.09.696240 Preprint at https://doi.org/10.64898/2026.01.09.696240 (2026).
