# Supplementary figures and images for: ThermiQuant™ AquaStream: A portable instrument for quantitative colorimetric isothermal nucleic acid amplification reactions in paper and tube formats
Source: PLoS One. 2026 May 15;21(5):e0348607. doi: 10.1371/journal.pone.0348607 (PMC13178971; doi:10.1371/journal.pone.0348607)

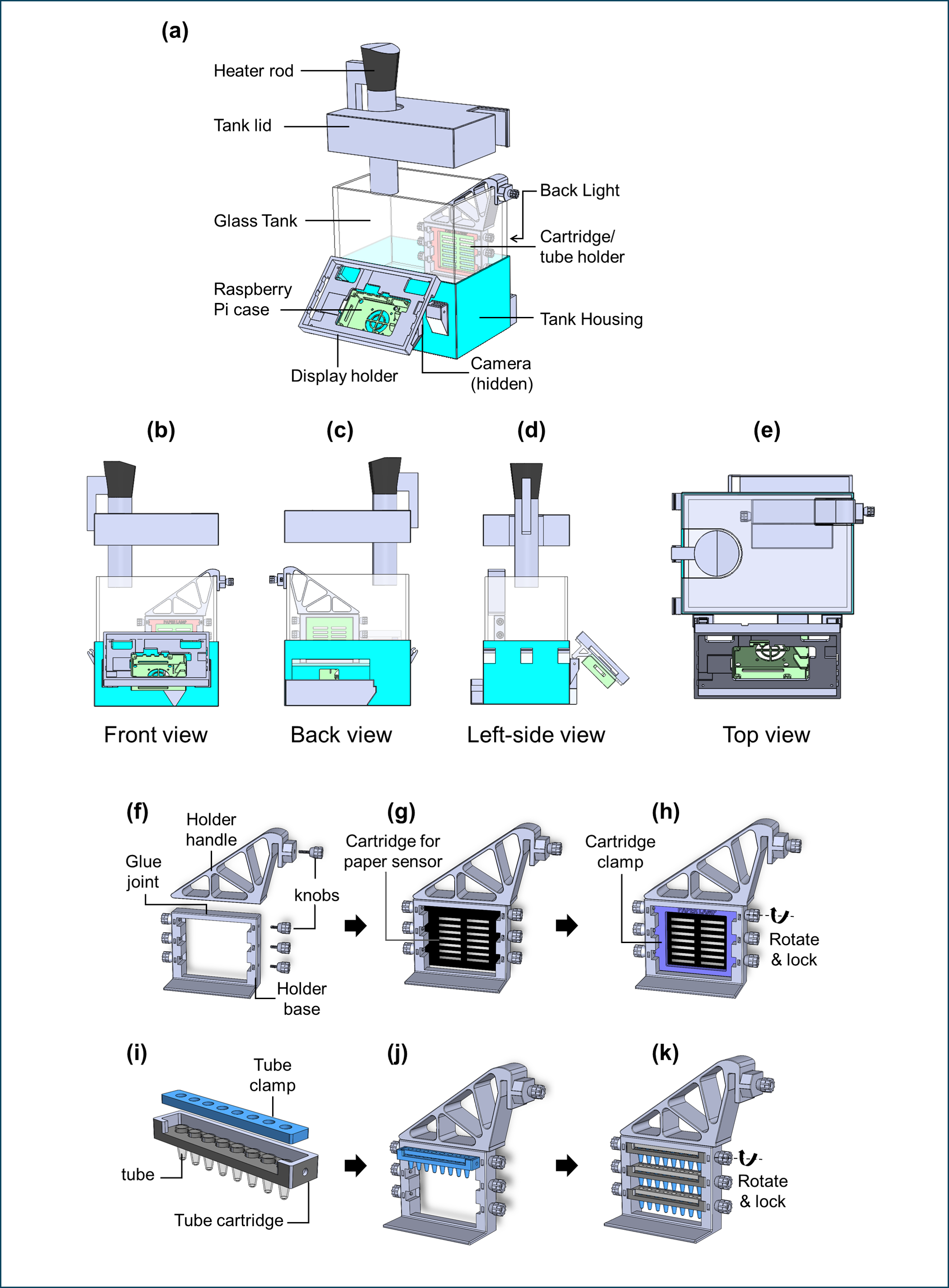

Supplement: S2 File — All CAD design files are included. (ZIP) [file pone.0348607.s002.zip › Design_files/Design_file_ref.png]

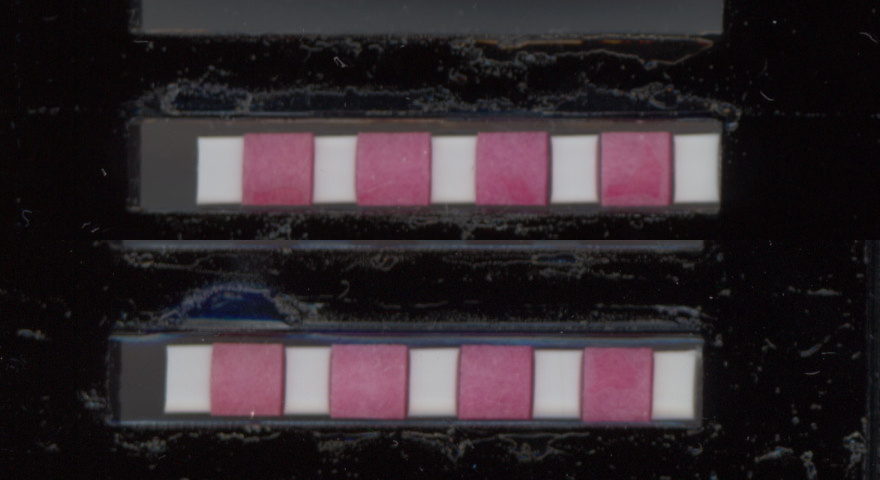

Supplement: S3 File — Amplimetrics™ software as well as ThermiQuant™ AquaStream source codes. (ZIP) [file pone.0348607.s003.zip › Software/02_Amplimetrics-V1.2/test_data/Timelapse_Image01.jpg]

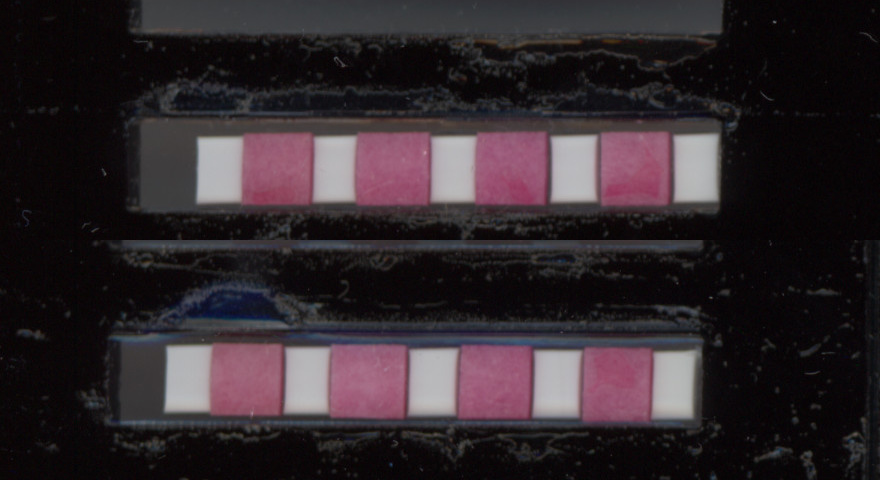

Supplement: S3 File — Amplimetrics™ software as well as ThermiQuant™ AquaStream source codes. (ZIP) [file pone.0348607.s003.zip › Software/02_Amplimetrics-V1.2/test_data/Timelapse_Image02.jpg]

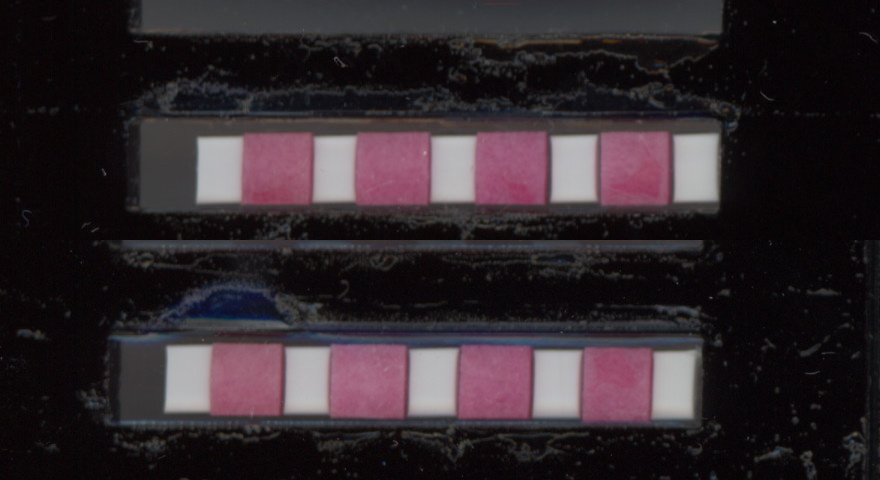

Supplement: S3 File — Amplimetrics™ software as well as ThermiQuant™ AquaStream source codes. (ZIP) [file pone.0348607.s003.zip › Software/02_Amplimetrics-V1.2/test_data/Timelapse_Image03.jpg]

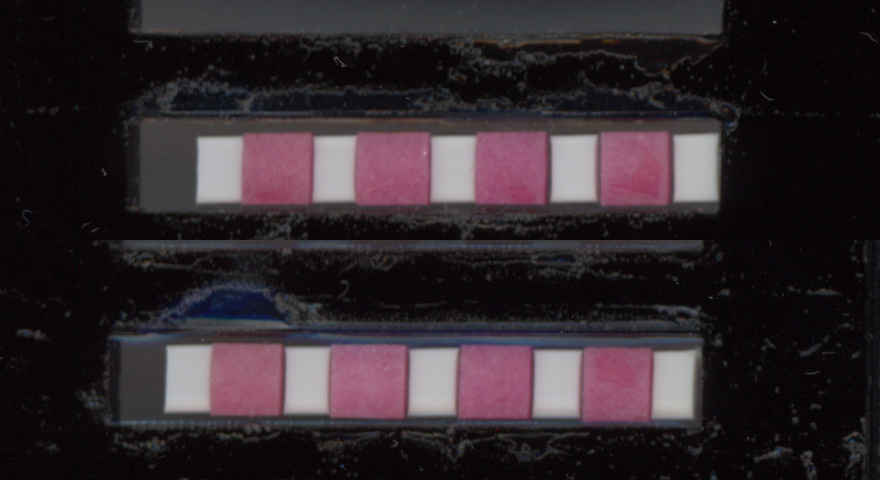

Supplement: S3 File — Amplimetrics™ software as well as ThermiQuant™ AquaStream source codes. (ZIP) [file pone.0348607.s003.zip › Software/02_Amplimetrics-V1.2/test_data/Timelapse_Image04.jpg]

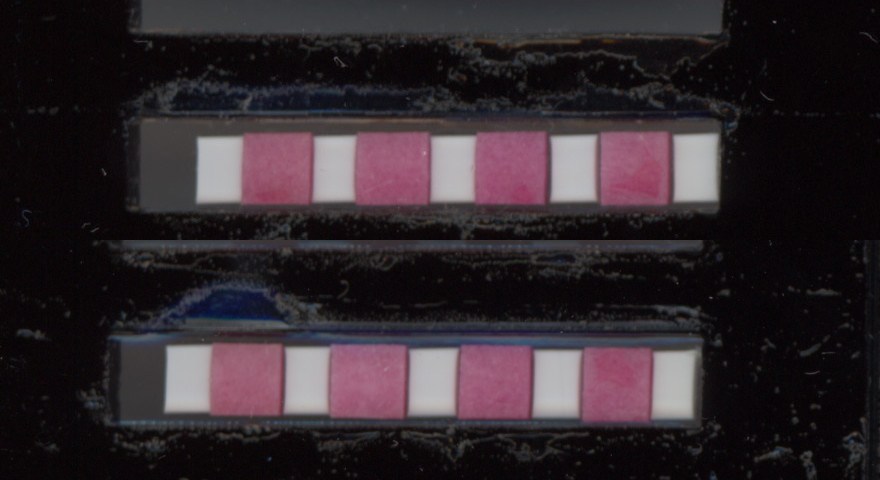

Supplement: S3 File — Amplimetrics™ software as well as ThermiQuant™ AquaStream source codes. (ZIP) [file pone.0348607.s003.zip › Software/02_Amplimetrics-V1.2/test_data/Timelapse_Image05.jpg]

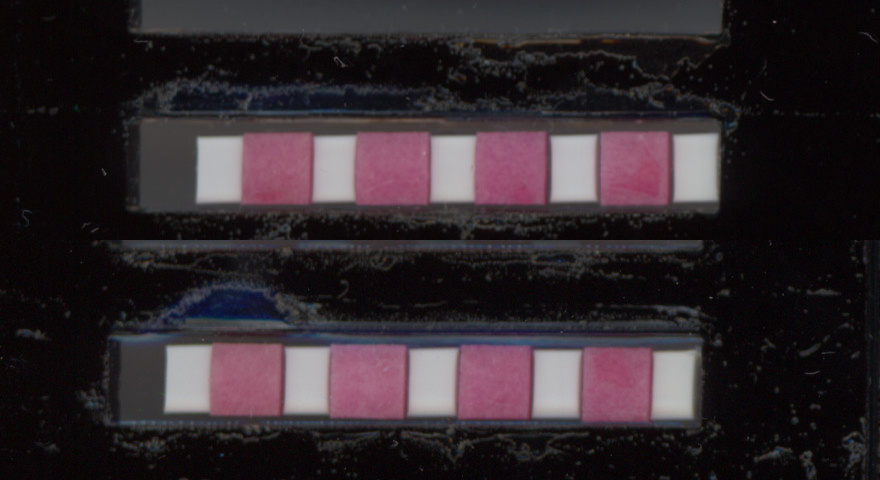

Supplement: S3 File — Amplimetrics™ software as well as ThermiQuant™ AquaStream source codes. (ZIP) [file pone.0348607.s003.zip › Software/02_Amplimetrics-V1.2/test_data/Timelapse_Image06.jpg]

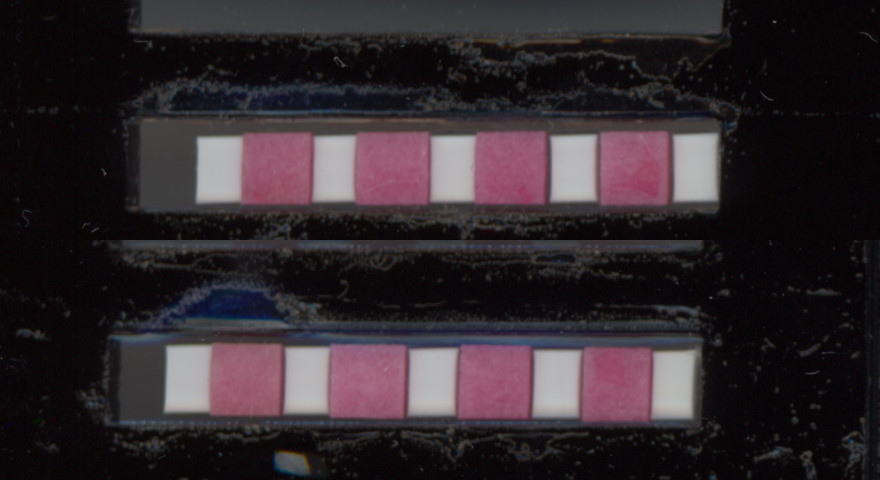

Supplement: S3 File — Amplimetrics™ software as well as ThermiQuant™ AquaStream source codes. (ZIP) [file pone.0348607.s003.zip › Software/02_Amplimetrics-V1.2/test_data/Timelapse_Image07.jpg]

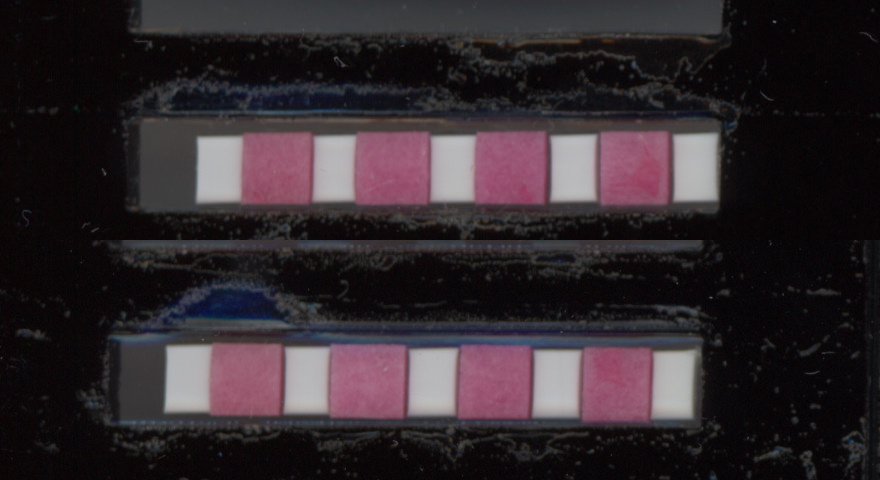

Supplement: S3 File — Amplimetrics™ software as well as ThermiQuant™ AquaStream source codes. (ZIP) [file pone.0348607.s003.zip › Software/02_Amplimetrics-V1.2/test_data/Timelapse_Image08.jpg]

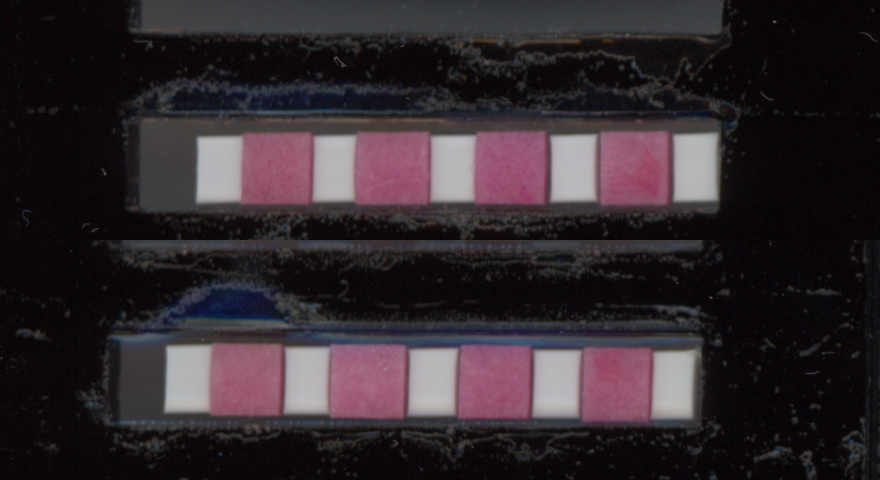

Supplement: S3 File — Amplimetrics™ software as well as ThermiQuant™ AquaStream source codes. (ZIP) [file pone.0348607.s003.zip › Software/02_Amplimetrics-V1.2/test_data/Timelapse_Image09.jpg]

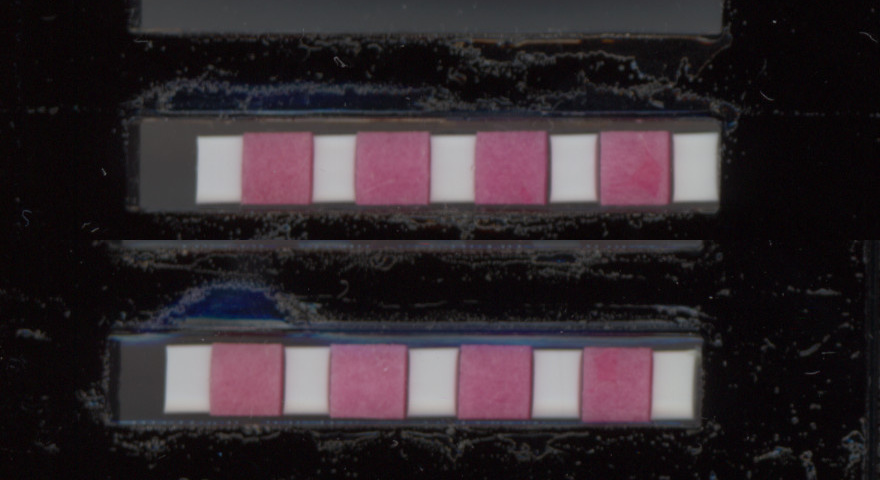

Supplement: S3 File — Amplimetrics™ software as well as ThermiQuant™ AquaStream source codes. (ZIP) [file pone.0348607.s003.zip › Software/02_Amplimetrics-V1.2/test_data/Timelapse_Image10.jpg]

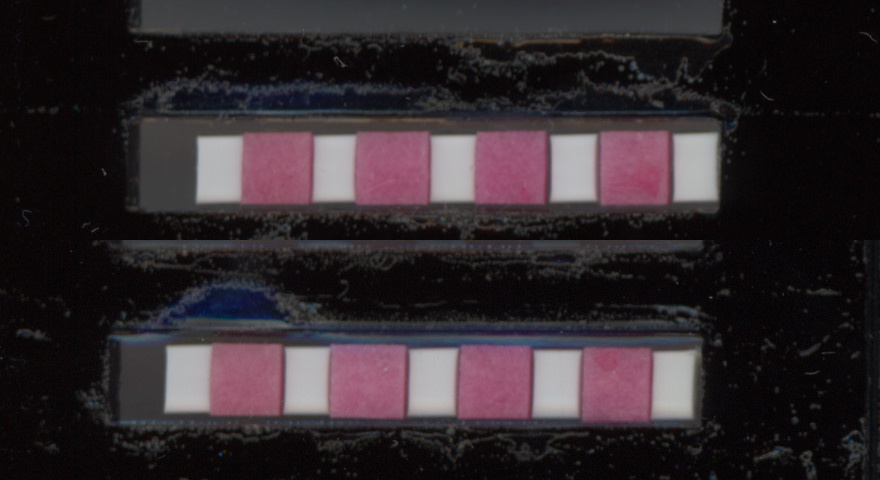

Supplement: S3 File — Amplimetrics™ software as well as ThermiQuant™ AquaStream source codes. (ZIP) [file pone.0348607.s003.zip › Software/02_Amplimetrics-V1.2/test_data/Timelapse_Image11.jpg]

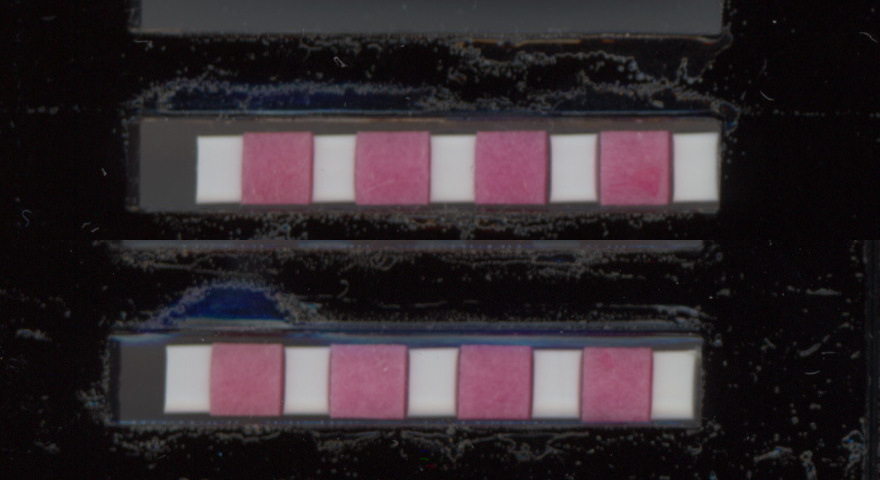

Supplement: S3 File — Amplimetrics™ software as well as ThermiQuant™ AquaStream source codes. (ZIP) [file pone.0348607.s003.zip › Software/02_Amplimetrics-V1.2/test_data/Timelapse_Image12.jpg]

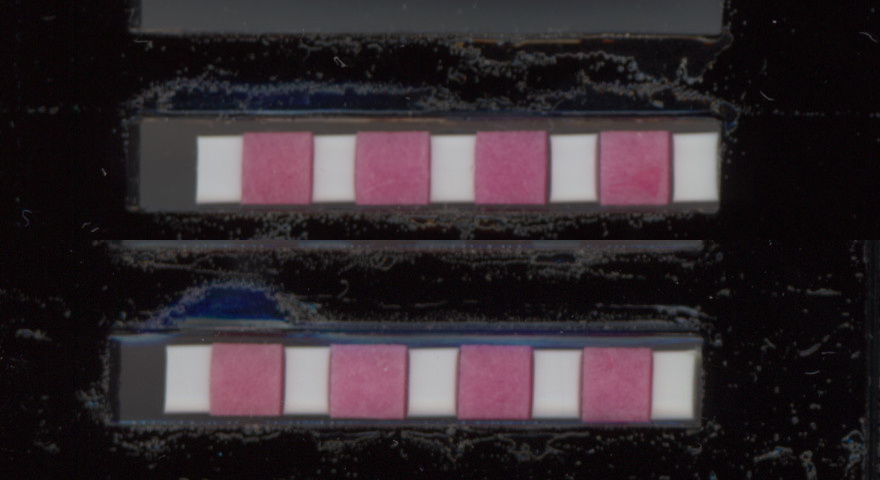

Supplement: S3 File — Amplimetrics™ software as well as ThermiQuant™ AquaStream source codes. (ZIP) [file pone.0348607.s003.zip › Software/02_Amplimetrics-V1.2/test_data/Timelapse_Image13.jpg]

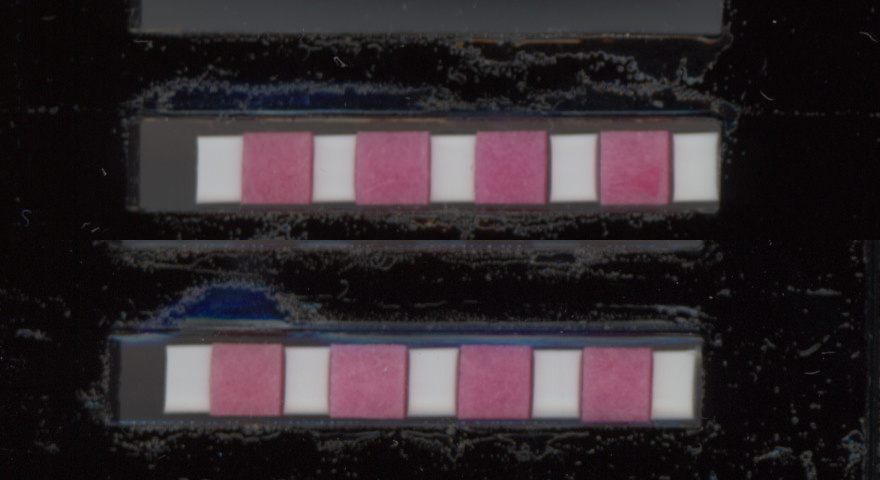

Supplement: S3 File — Amplimetrics™ software as well as ThermiQuant™ AquaStream source codes. (ZIP) [file pone.0348607.s003.zip › Software/02_Amplimetrics-V1.2/test_data/Timelapse_Image14.jpg]

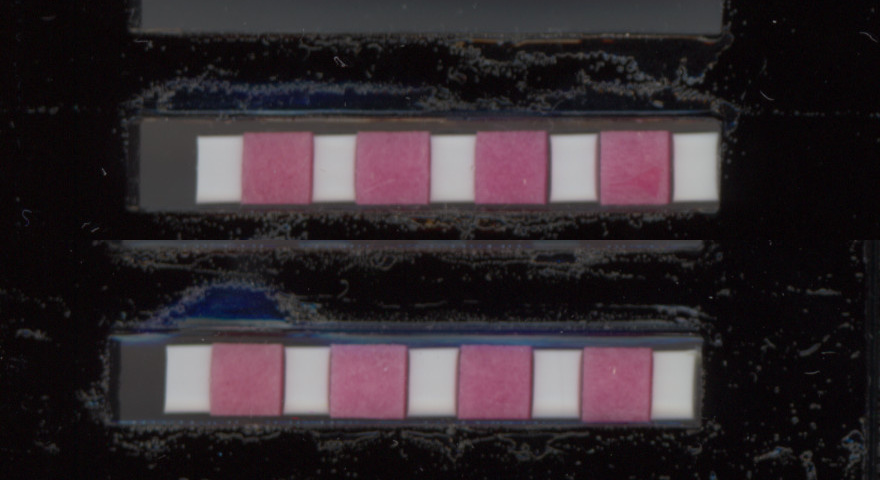

Supplement: S3 File — Amplimetrics™ software as well as ThermiQuant™ AquaStream source codes. (ZIP) [file pone.0348607.s003.zip › Software/02_Amplimetrics-V1.2/test_data/Timelapse_Image15.jpg]

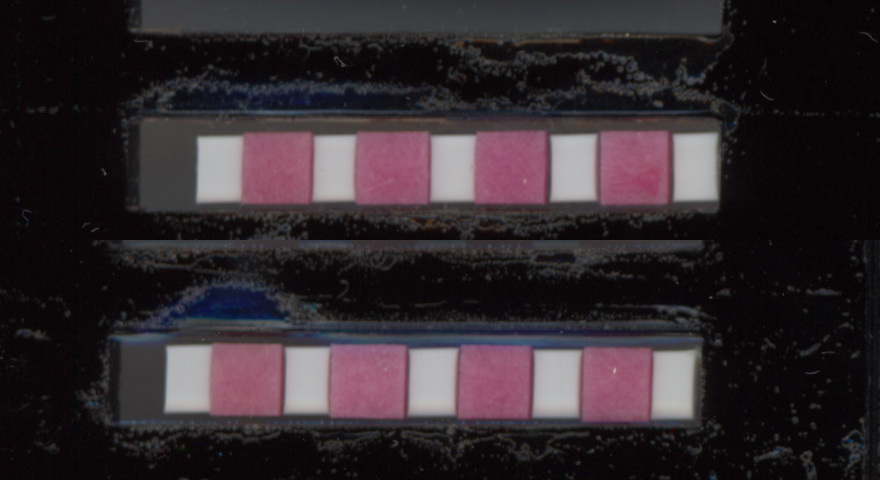

Supplement: S3 File — Amplimetrics™ software as well as ThermiQuant™ AquaStream source codes. (ZIP) [file pone.0348607.s003.zip › Software/02_Amplimetrics-V1.2/test_data/Timelapse_Image16.jpg]

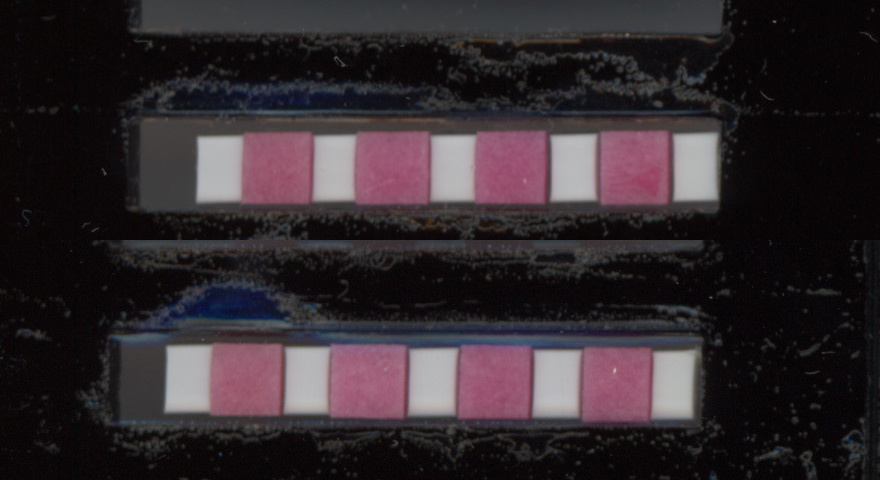

Supplement: S3 File — Amplimetrics™ software as well as ThermiQuant™ AquaStream source codes. (ZIP) [file pone.0348607.s003.zip › Software/02_Amplimetrics-V1.2/test_data/Timelapse_Image17.jpg]

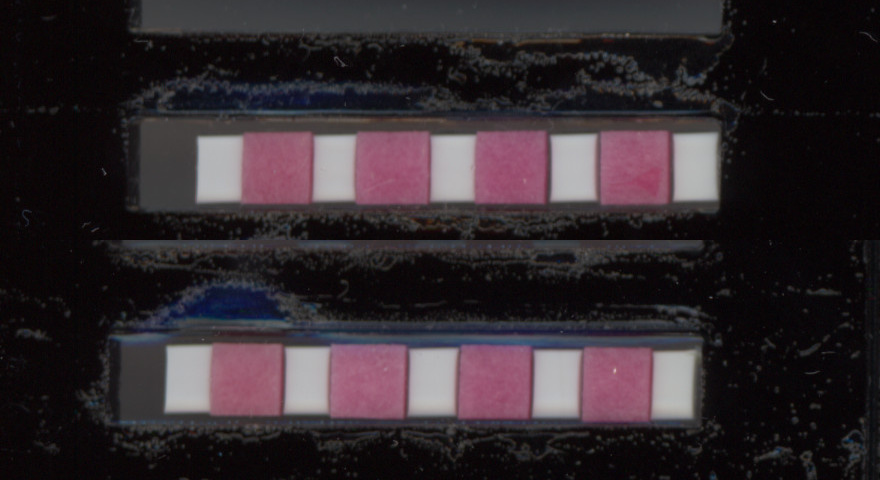

Supplement: S3 File — Amplimetrics™ software as well as ThermiQuant™ AquaStream source codes. (ZIP) [file pone.0348607.s003.zip › Software/02_Amplimetrics-V1.2/test_data/Timelapse_Image18.jpg]

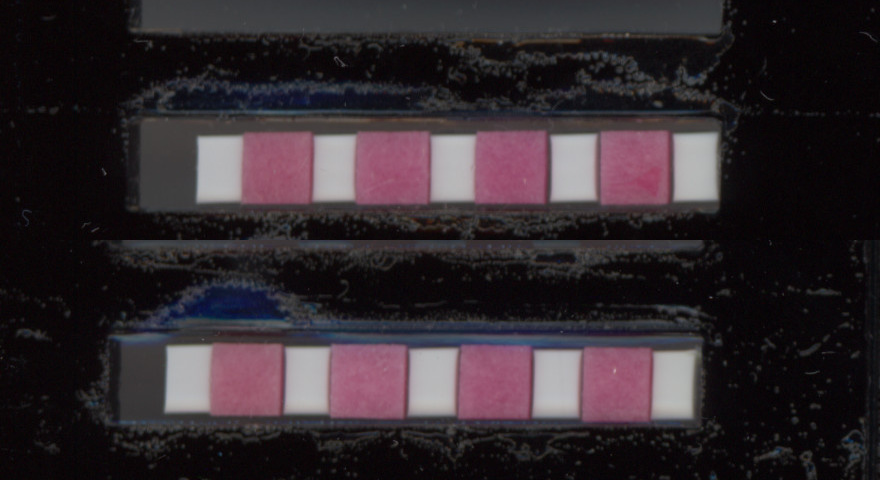

Supplement: S3 File — Amplimetrics™ software as well as ThermiQuant™ AquaStream source codes. (ZIP) [file pone.0348607.s003.zip › Software/02_Amplimetrics-V1.2/test_data/Timelapse_Image19.jpg]

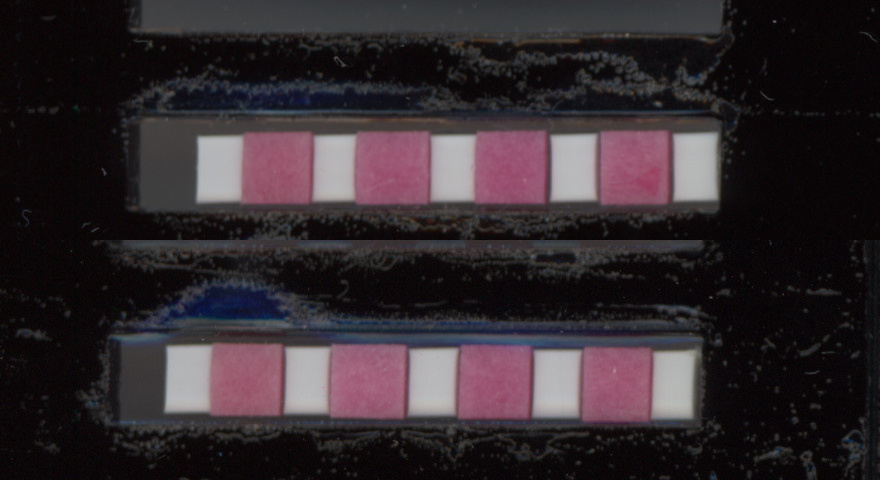

Supplement: S3 File — Amplimetrics™ software as well as ThermiQuant™ AquaStream source codes. (ZIP) [file pone.0348607.s003.zip › Software/02_Amplimetrics-V1.2/test_data/Timelapse_Image20.jpg]

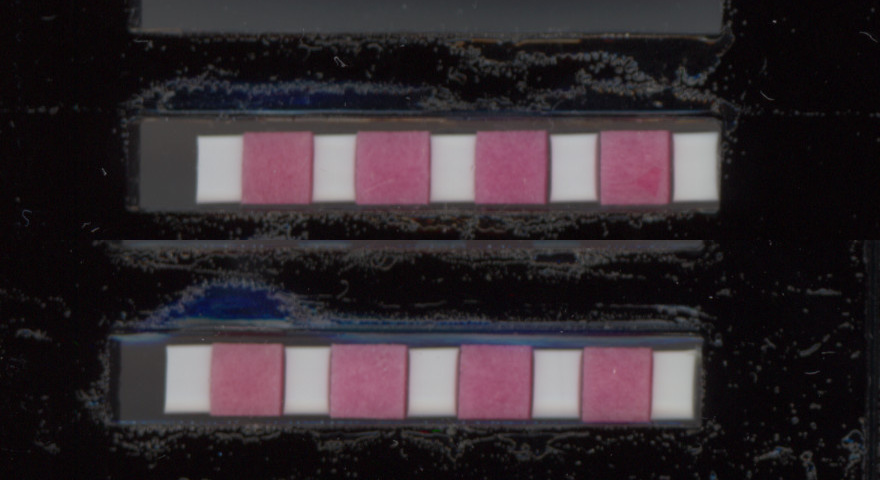

Supplement: S3 File — Amplimetrics™ software as well as ThermiQuant™ AquaStream source codes. (ZIP) [file pone.0348607.s003.zip › Software/02_Amplimetrics-V1.2/test_data/Timelapse_Image21.jpg]

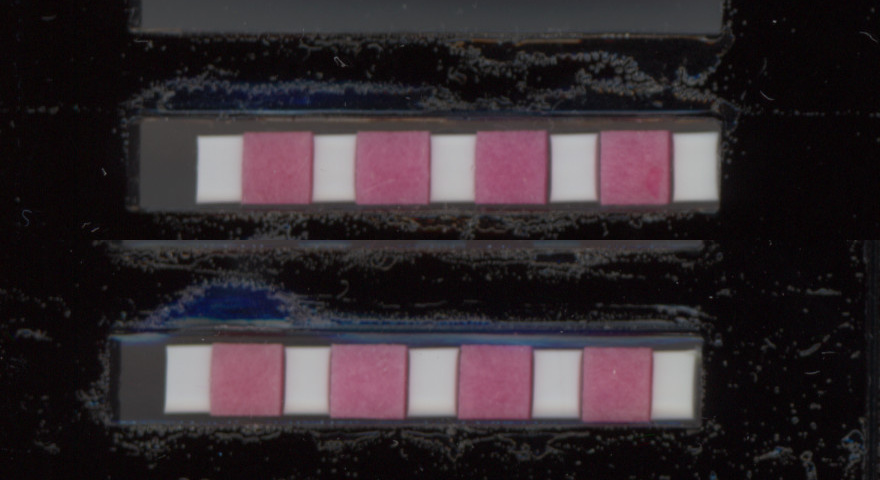

Supplement: S3 File — Amplimetrics™ software as well as ThermiQuant™ AquaStream source codes. (ZIP) [file pone.0348607.s003.zip › Software/02_Amplimetrics-V1.2/test_data/Timelapse_Image22.jpg]

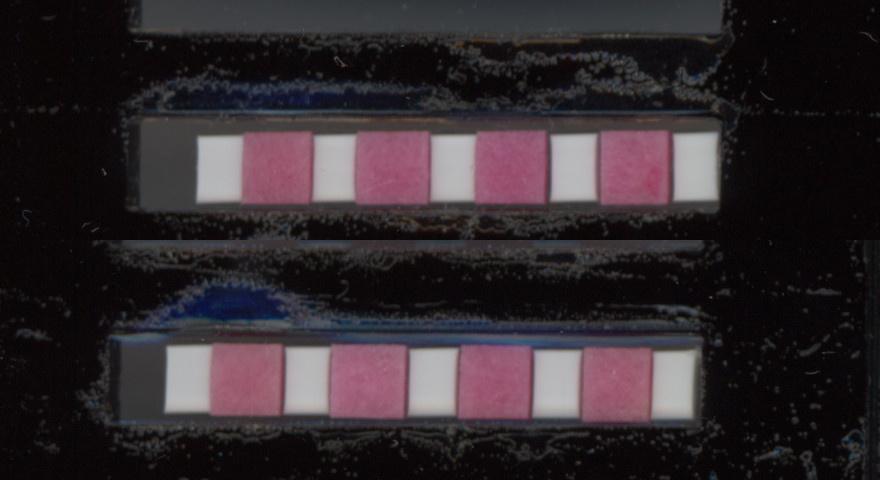

Supplement: S3 File — Amplimetrics™ software as well as ThermiQuant™ AquaStream source codes. (ZIP) [file pone.0348607.s003.zip › Software/02_Amplimetrics-V1.2/test_data/Timelapse_Image23.jpg]

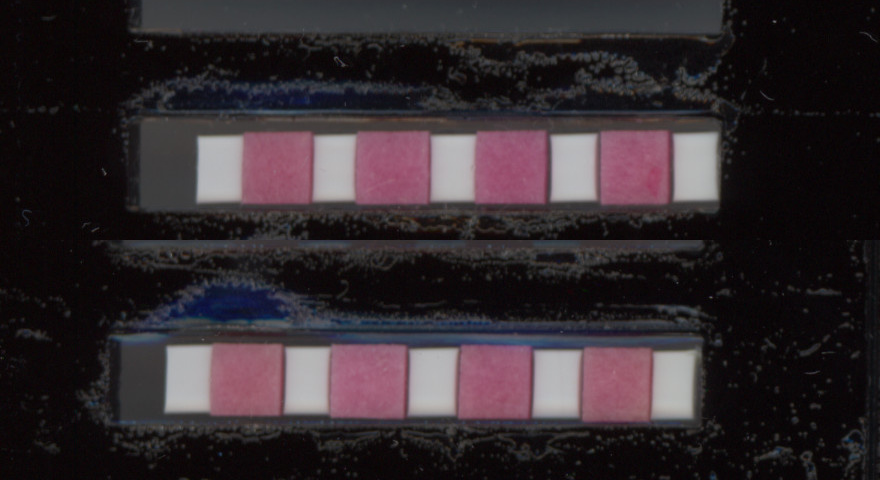

Supplement: S3 File — Amplimetrics™ software as well as ThermiQuant™ AquaStream source codes. (ZIP) [file pone.0348607.s003.zip › Software/02_Amplimetrics-V1.2/test_data/Timelapse_Image24.jpg]

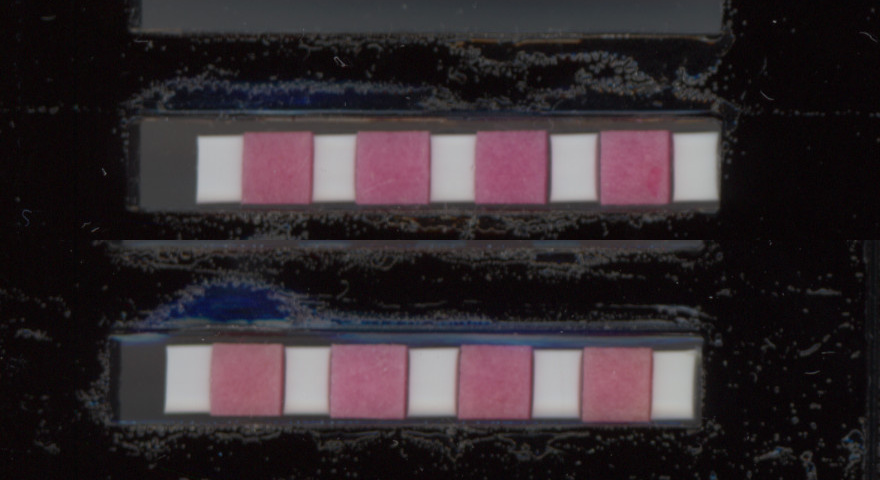

Supplement: S3 File — Amplimetrics™ software as well as ThermiQuant™ AquaStream source codes. (ZIP) [file pone.0348607.s003.zip › Software/02_Amplimetrics-V1.2/test_data/Timelapse_Image25.jpg]

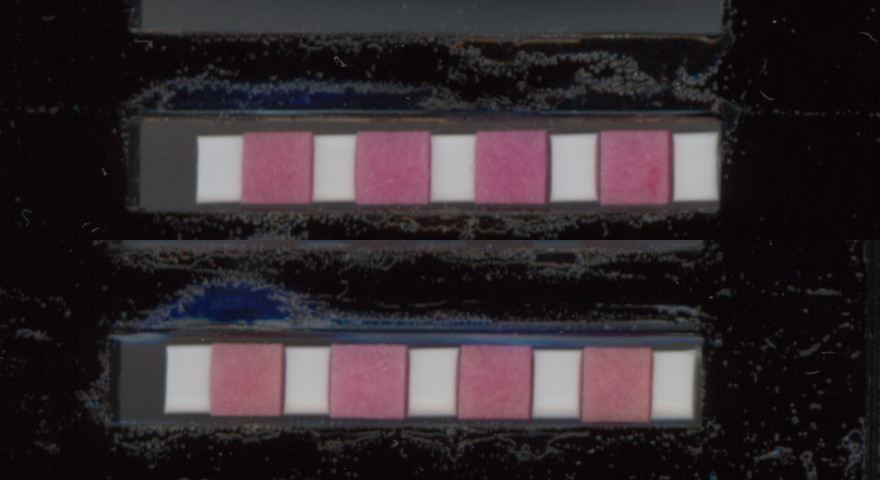

Supplement: S3 File — Amplimetrics™ software as well as ThermiQuant™ AquaStream source codes. (ZIP) [file pone.0348607.s003.zip › Software/02_Amplimetrics-V1.2/test_data/Timelapse_Image26.jpg]

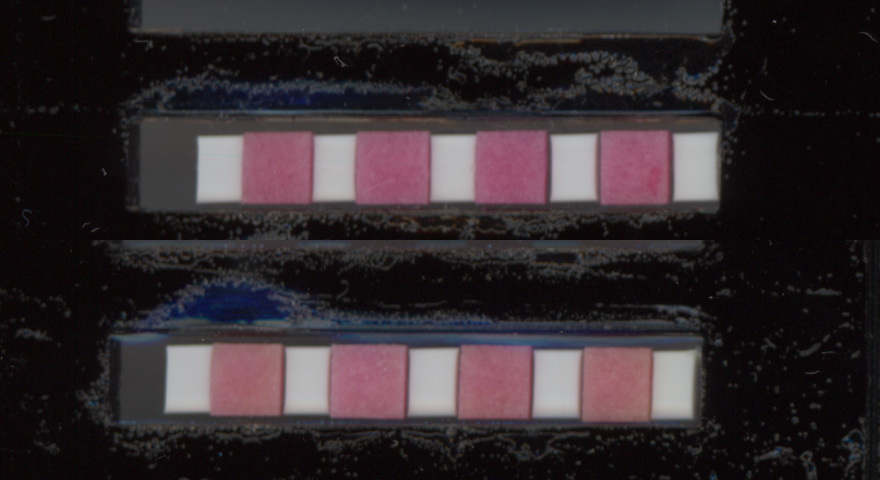

Supplement: S3 File — Amplimetrics™ software as well as ThermiQuant™ AquaStream source codes. (ZIP) [file pone.0348607.s003.zip › Software/02_Amplimetrics-V1.2/test_data/Timelapse_Image27.jpg]

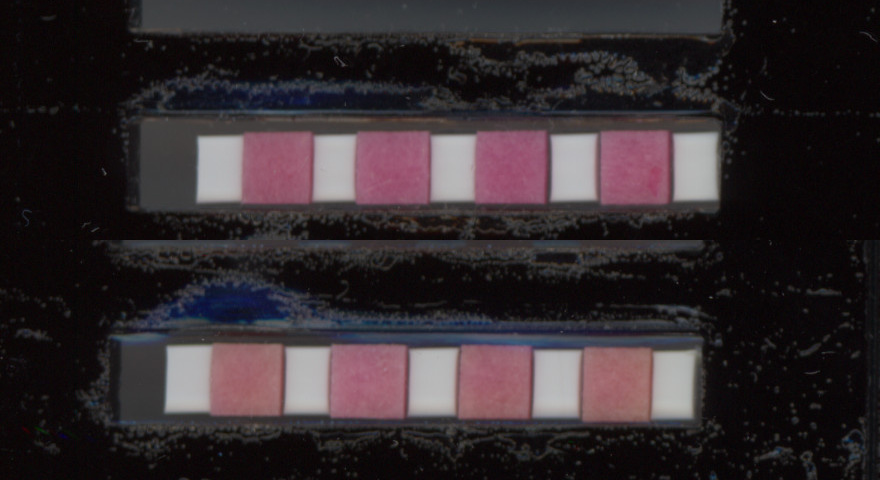

Supplement: S3 File — Amplimetrics™ software as well as ThermiQuant™ AquaStream source codes. (ZIP) [file pone.0348607.s003.zip › Software/02_Amplimetrics-V1.2/test_data/Timelapse_Image28.jpg]

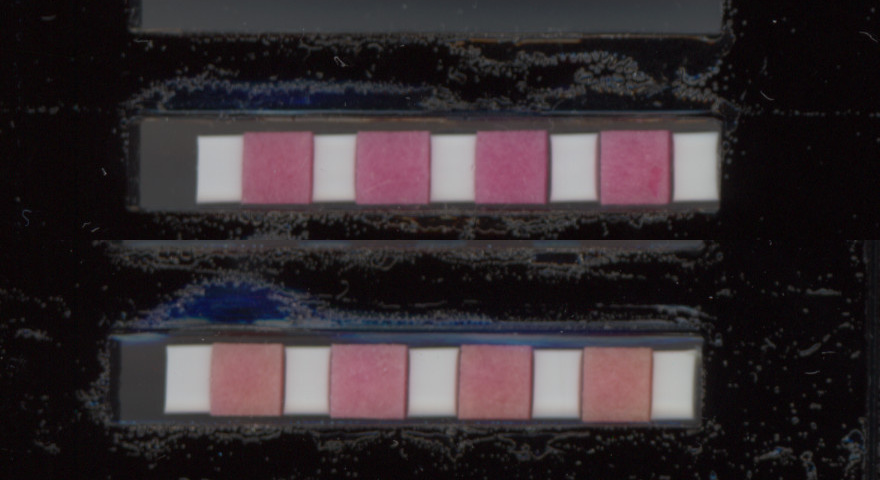

Supplement: S3 File — Amplimetrics™ software as well as ThermiQuant™ AquaStream source codes. (ZIP) [file pone.0348607.s003.zip › Software/02_Amplimetrics-V1.2/test_data/Timelapse_Image29.jpg]

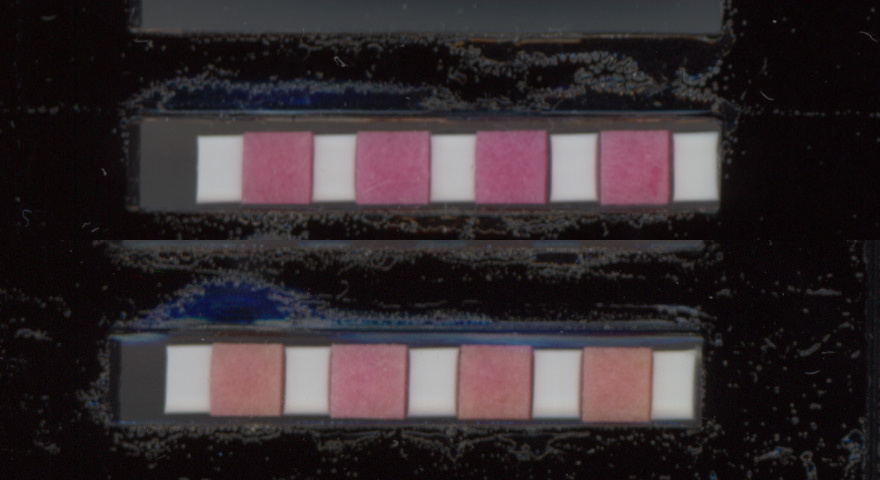

Supplement: S3 File — Amplimetrics™ software as well as ThermiQuant™ AquaStream source codes. (ZIP) [file pone.0348607.s003.zip › Software/02_Amplimetrics-V1.2/test_data/Timelapse_Image30.jpg]

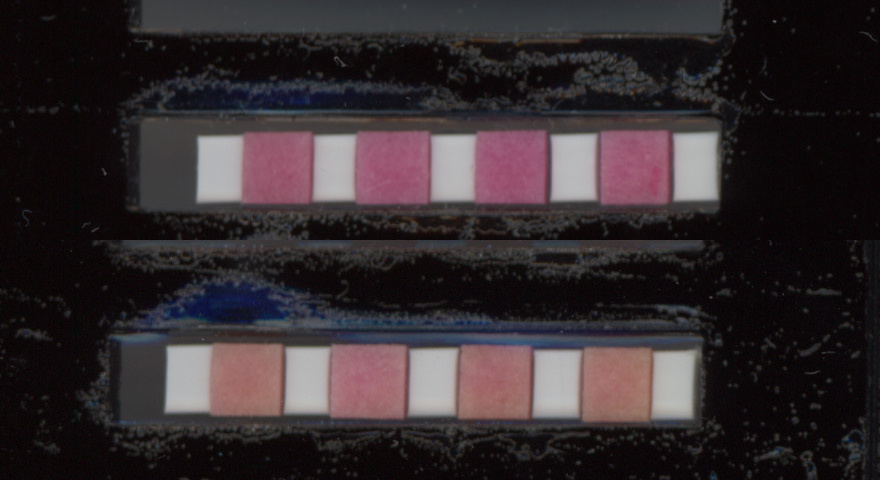

Supplement: S3 File — Amplimetrics™ software as well as ThermiQuant™ AquaStream source codes. (ZIP) [file pone.0348607.s003.zip › Software/02_Amplimetrics-V1.2/test_data/Timelapse_Image31.jpg]

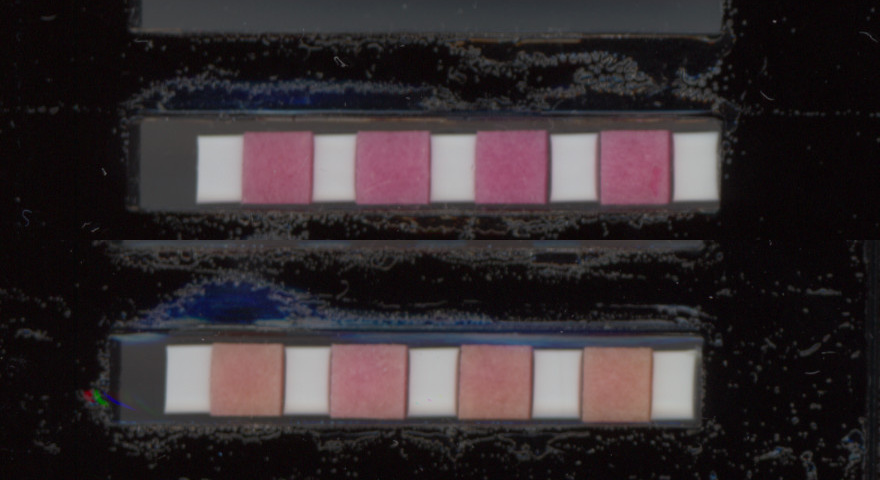

Supplement: S3 File — Amplimetrics™ software as well as ThermiQuant™ AquaStream source codes. (ZIP) [file pone.0348607.s003.zip › Software/02_Amplimetrics-V1.2/test_data/Timelapse_Image32.jpg]

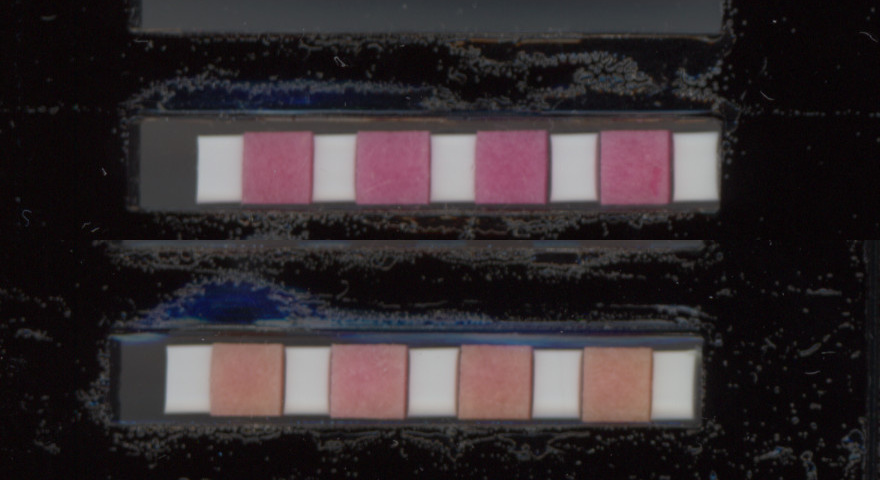

Supplement: S3 File — Amplimetrics™ software as well as ThermiQuant™ AquaStream source codes. (ZIP) [file pone.0348607.s003.zip › Software/02_Amplimetrics-V1.2/test_data/Timelapse_Image33.jpg]

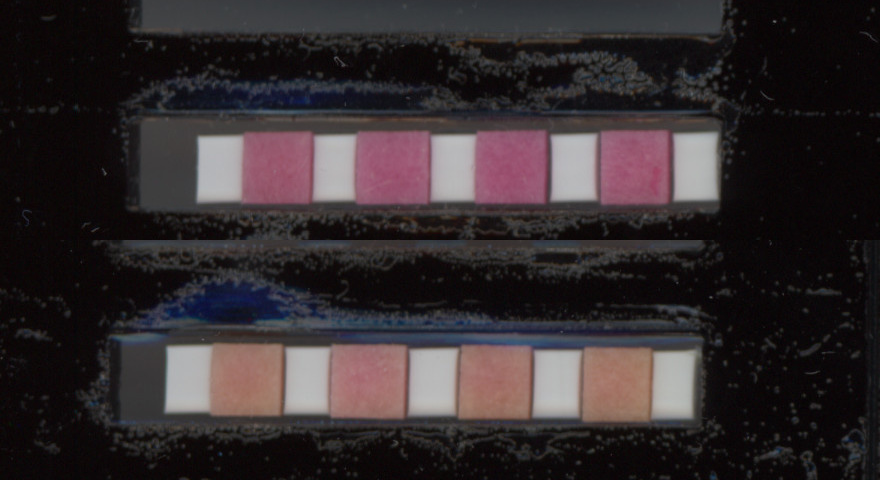

Supplement: S3 File — Amplimetrics™ software as well as ThermiQuant™ AquaStream source codes. (ZIP) [file pone.0348607.s003.zip › Software/02_Amplimetrics-V1.2/test_data/Timelapse_Image34.jpg]

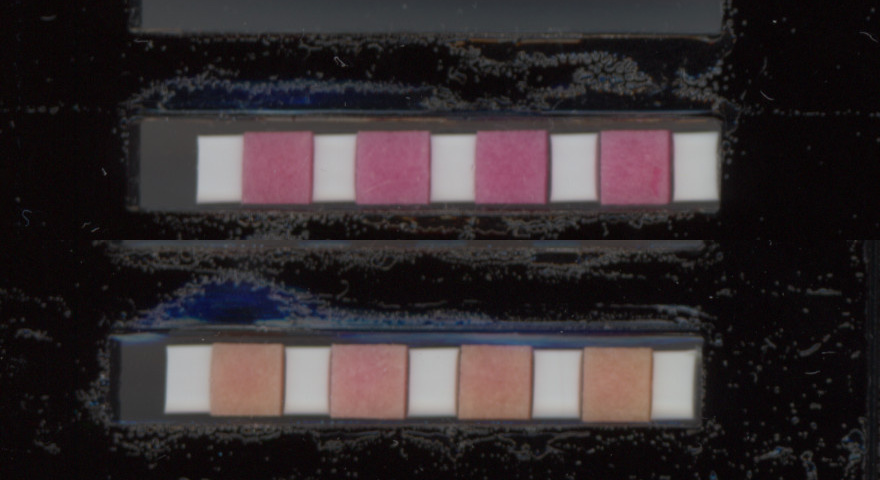

Supplement: S3 File — Amplimetrics™ software as well as ThermiQuant™ AquaStream source codes. (ZIP) [file pone.0348607.s003.zip › Software/02_Amplimetrics-V1.2/test_data/Timelapse_Image35.jpg]

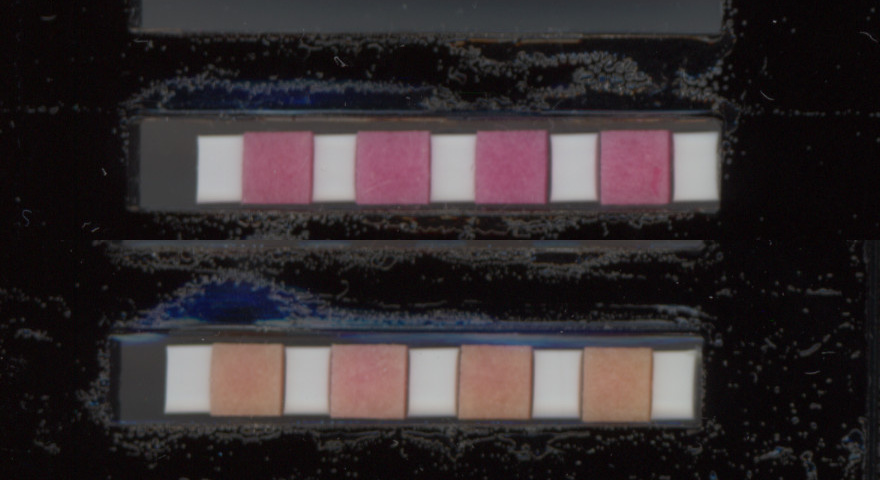

Supplement: S3 File — Amplimetrics™ software as well as ThermiQuant™ AquaStream source codes. (ZIP) [file pone.0348607.s003.zip › Software/02_Amplimetrics-V1.2/test_data/Timelapse_Image36.jpg]

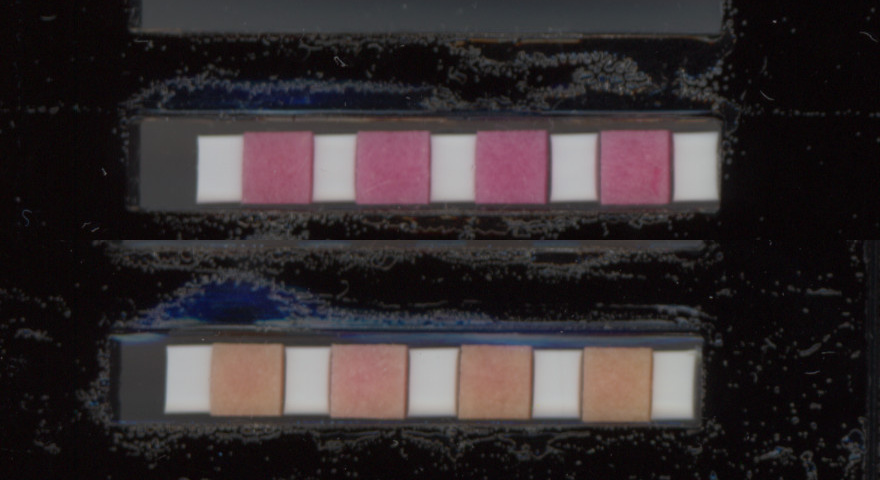

Supplement: S3 File — Amplimetrics™ software as well as ThermiQuant™ AquaStream source codes. (ZIP) [file pone.0348607.s003.zip › Software/02_Amplimetrics-V1.2/test_data/Timelapse_Image37.jpg]

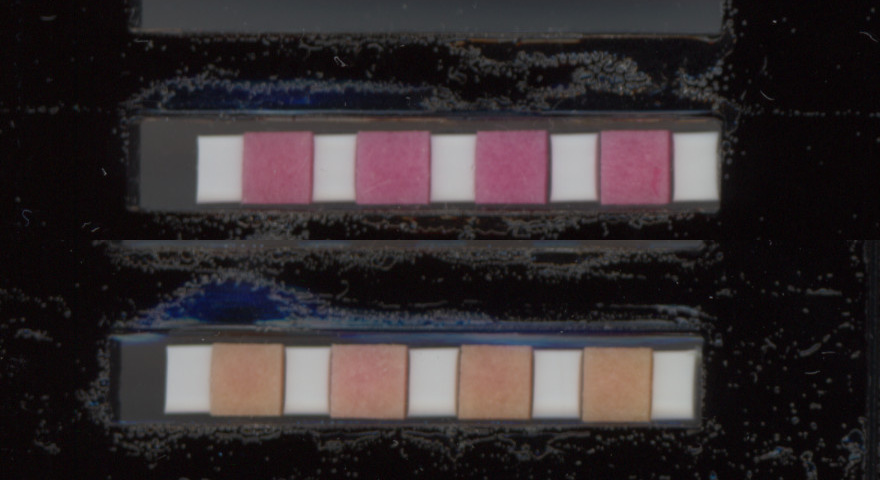

Supplement: S3 File — Amplimetrics™ software as well as ThermiQuant™ AquaStream source codes. (ZIP) [file pone.0348607.s003.zip › Software/02_Amplimetrics-V1.2/test_data/Timelapse_Image38.jpg]

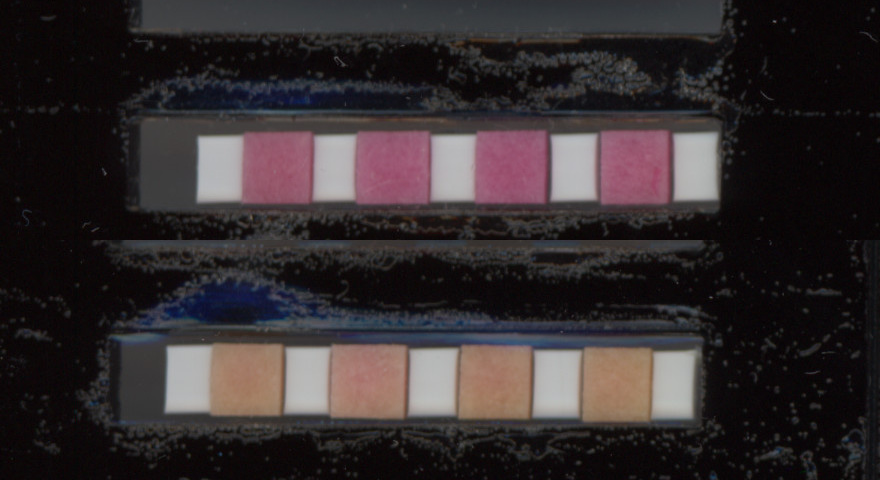

Supplement: S3 File — Amplimetrics™ software as well as ThermiQuant™ AquaStream source codes. (ZIP) [file pone.0348607.s003.zip › Software/02_Amplimetrics-V1.2/test_data/Timelapse_Image39.jpg]

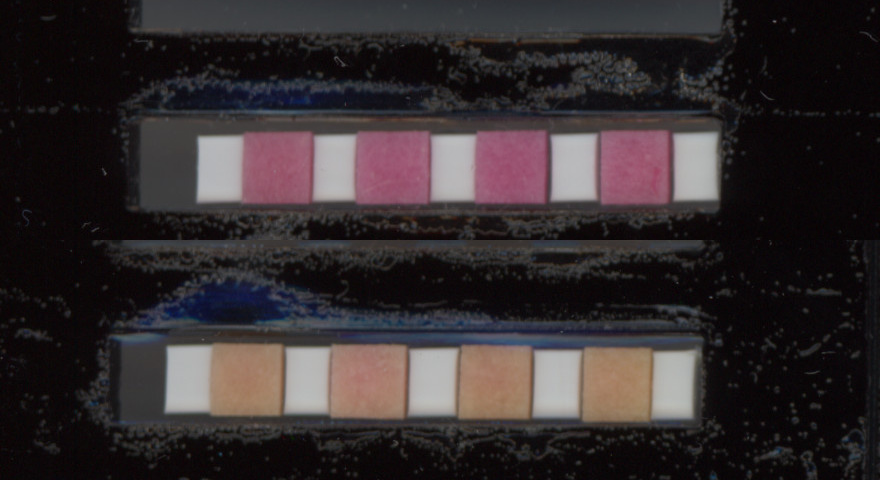

Supplement: S3 File — Amplimetrics™ software as well as ThermiQuant™ AquaStream source codes. (ZIP) [file pone.0348607.s003.zip › Software/02_Amplimetrics-V1.2/test_data/Timelapse_Image40.jpg]

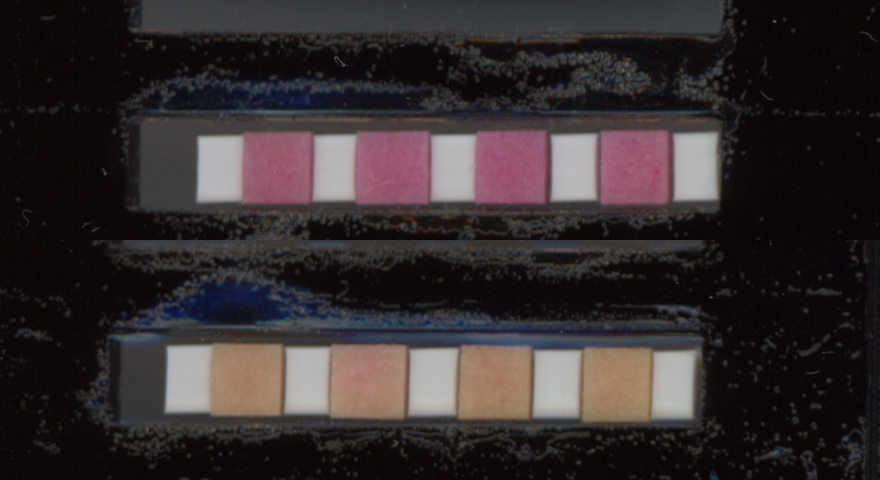

Supplement: S3 File — Amplimetrics™ software as well as ThermiQuant™ AquaStream source codes. (ZIP) [file pone.0348607.s003.zip › Software/02_Amplimetrics-V1.2/test_data/Timelapse_Image41.jpg]

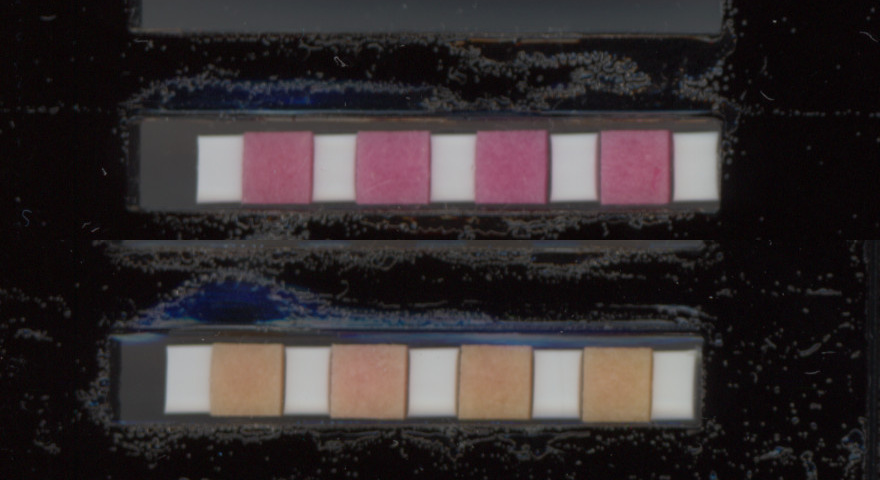

Supplement: S3 File — Amplimetrics™ software as well as ThermiQuant™ AquaStream source codes. (ZIP) [file pone.0348607.s003.zip › Software/02_Amplimetrics-V1.2/test_data/Timelapse_Image42.jpg]

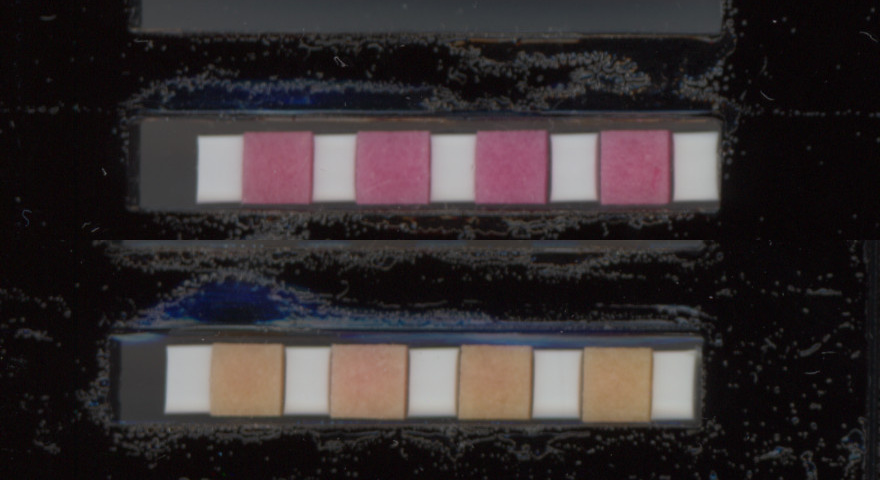

Supplement: S3 File — Amplimetrics™ software as well as ThermiQuant™ AquaStream source codes. (ZIP) [file pone.0348607.s003.zip › Software/02_Amplimetrics-V1.2/test_data/Timelapse_Image43.jpg]

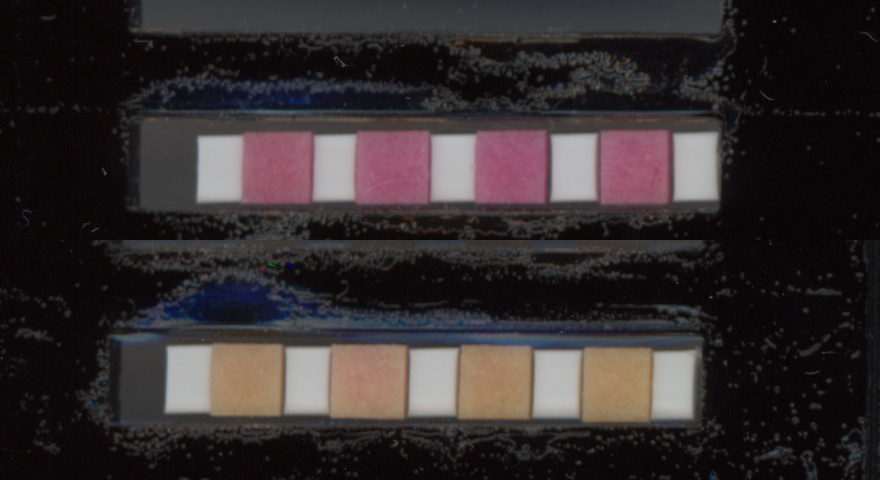

Supplement: S3 File — Amplimetrics™ software as well as ThermiQuant™ AquaStream source codes. (ZIP) [file pone.0348607.s003.zip › Software/02_Amplimetrics-V1.2/test_data/Timelapse_Image44.jpg]

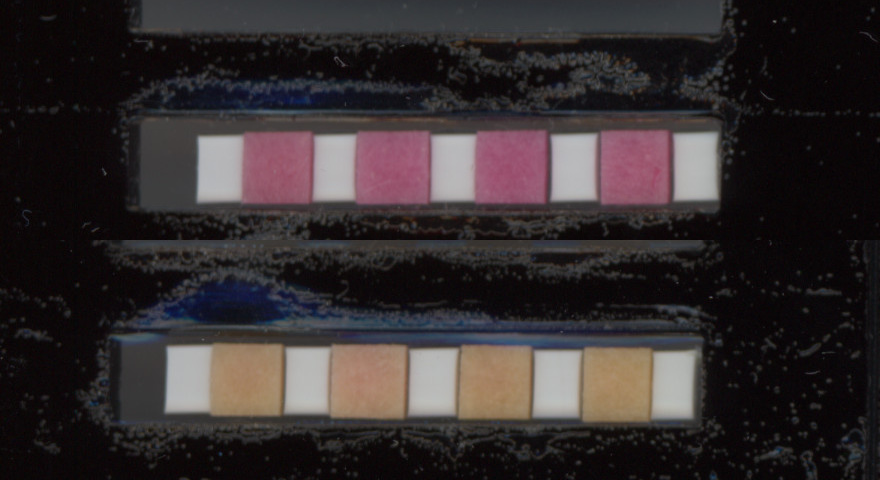

Supplement: S3 File — Amplimetrics™ software as well as ThermiQuant™ AquaStream source codes. (ZIP) [file pone.0348607.s003.zip › Software/02_Amplimetrics-V1.2/test_data/Timelapse_Image45.jpg]

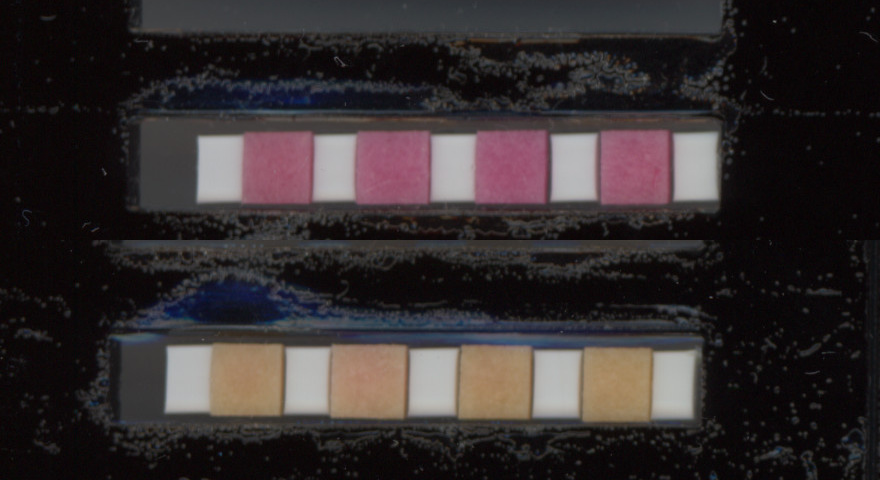

Supplement: S3 File — Amplimetrics™ software as well as ThermiQuant™ AquaStream source codes. (ZIP) [file pone.0348607.s003.zip › Software/02_Amplimetrics-V1.2/test_data/Timelapse_Image46.jpg]

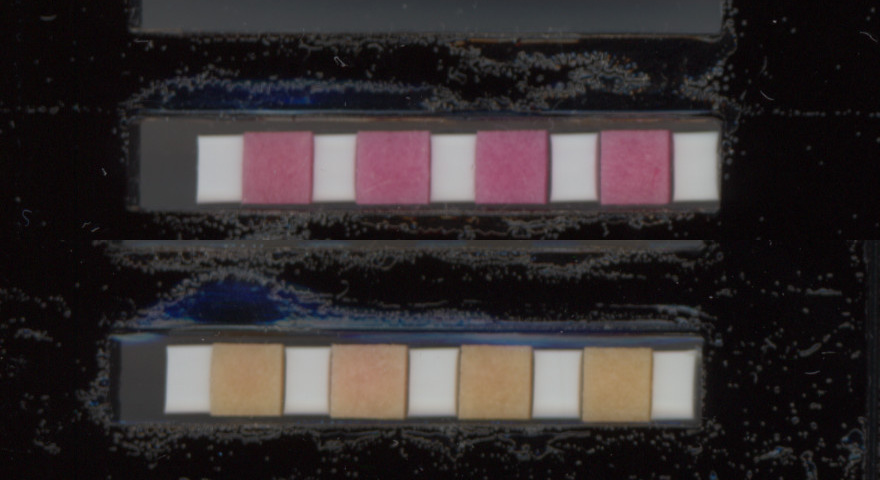

Supplement: S3 File — Amplimetrics™ software as well as ThermiQuant™ AquaStream source codes. (ZIP) [file pone.0348607.s003.zip › Software/02_Amplimetrics-V1.2/test_data/Timelapse_Image47.jpg]

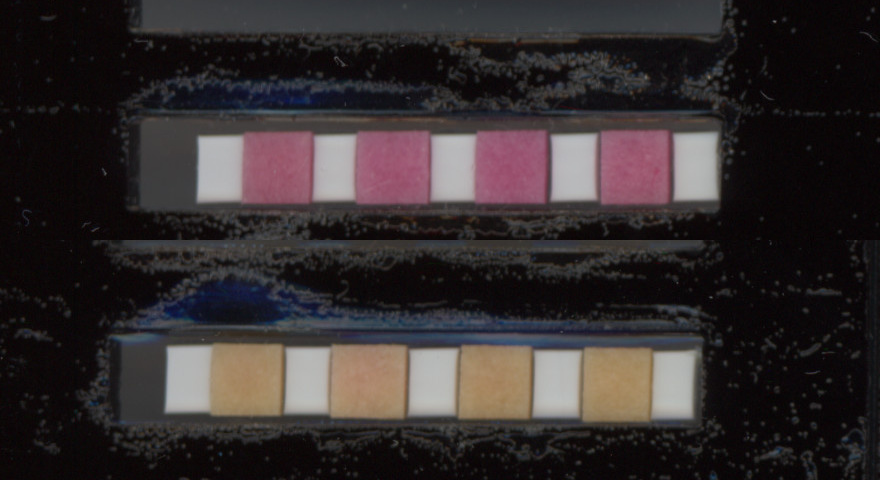

Supplement: S3 File — Amplimetrics™ software as well as ThermiQuant™ AquaStream source codes. (ZIP) [file pone.0348607.s003.zip › Software/02_Amplimetrics-V1.2/test_data/Timelapse_Image48.jpg]

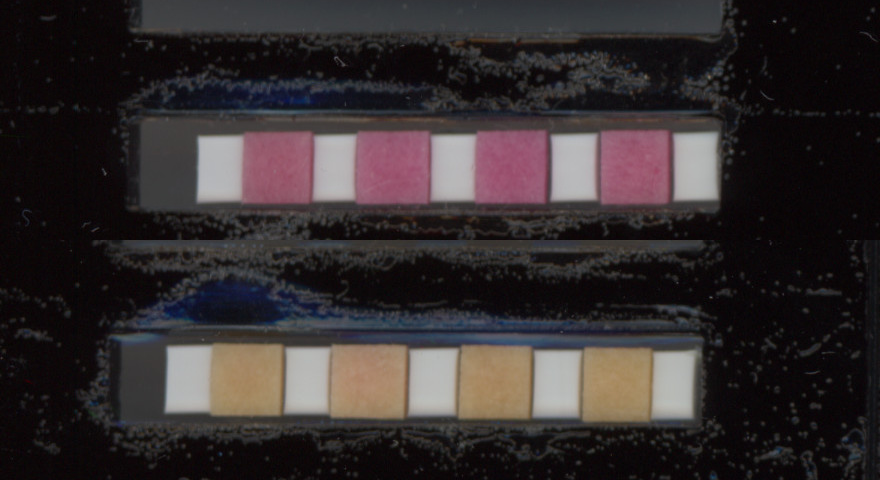

Supplement: S3 File — Amplimetrics™ software as well as ThermiQuant™ AquaStream source codes. (ZIP) [file pone.0348607.s003.zip › Software/02_Amplimetrics-V1.2/test_data/Timelapse_Image49.jpg]

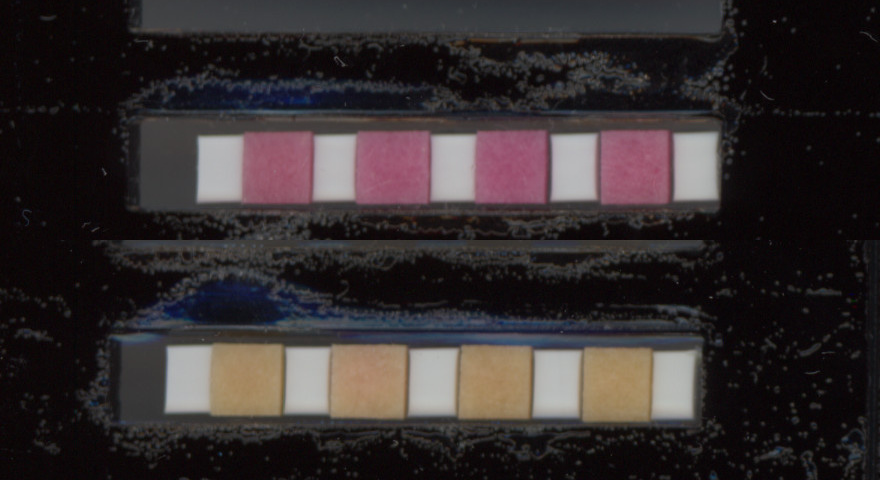

Supplement: S3 File — Amplimetrics™ software as well as ThermiQuant™ AquaStream source codes. (ZIP) [file pone.0348607.s003.zip › Software/02_Amplimetrics-V1.2/test_data/Timelapse_Image50.jpg]

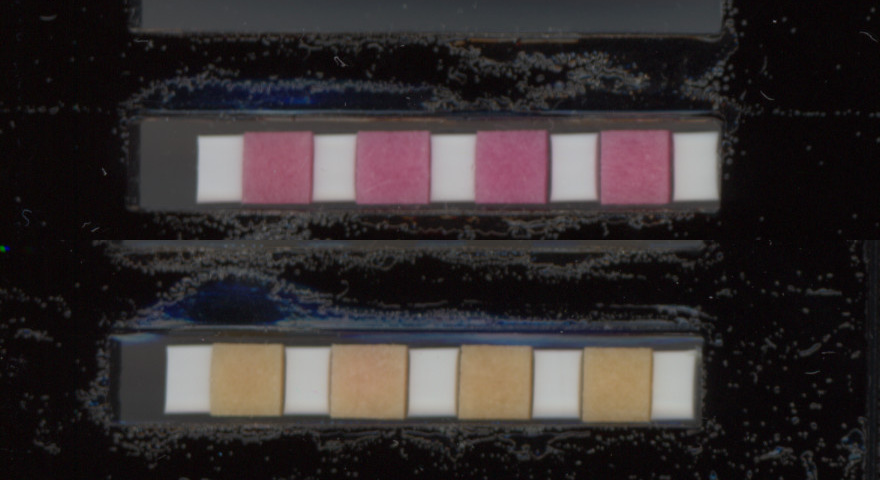

Supplement: S3 File — Amplimetrics™ software as well as ThermiQuant™ AquaStream source codes. (ZIP) [file pone.0348607.s003.zip › Software/02_Amplimetrics-V1.2/test_data/Timelapse_Image51.jpg]

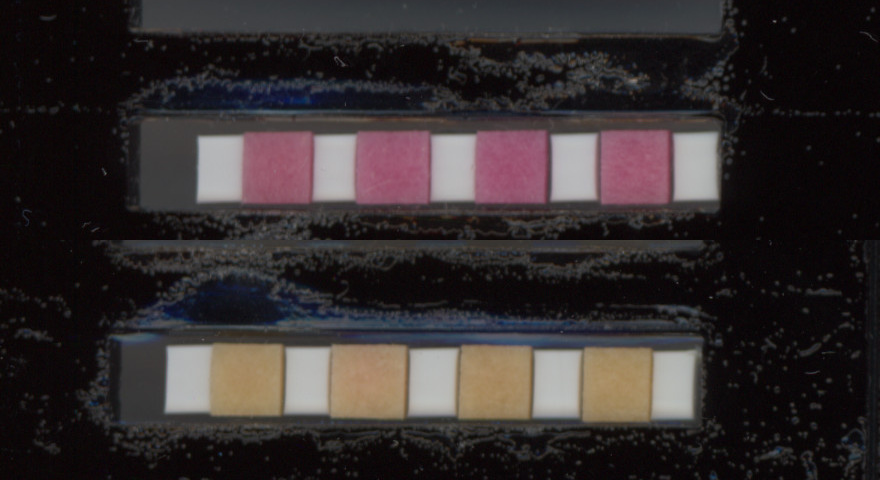

Supplement: S3 File — Amplimetrics™ software as well as ThermiQuant™ AquaStream source codes. (ZIP) [file pone.0348607.s003.zip › Software/02_Amplimetrics-V1.2/test_data/Timelapse_Image52.jpg]

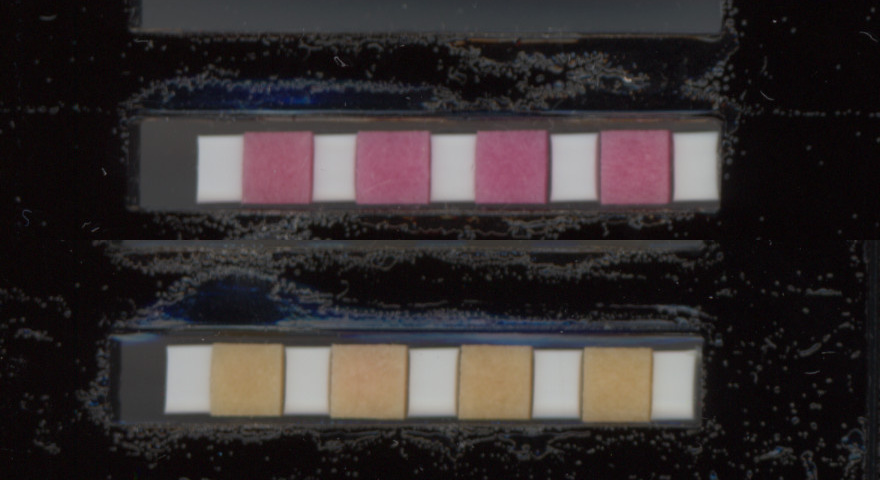

Supplement: S3 File — Amplimetrics™ software as well as ThermiQuant™ AquaStream source codes. (ZIP) [file pone.0348607.s003.zip › Software/02_Amplimetrics-V1.2/test_data/Timelapse_Image53.jpg]

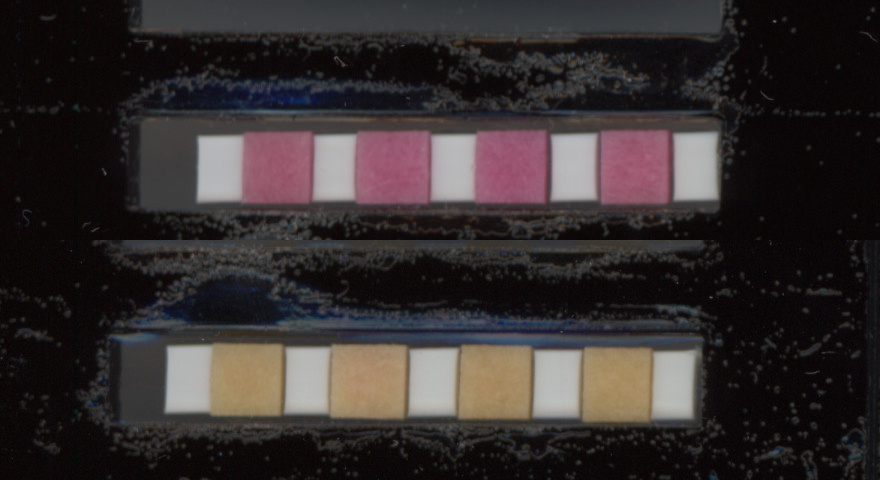

Supplement: S3 File — Amplimetrics™ software as well as ThermiQuant™ AquaStream source codes. (ZIP) [file pone.0348607.s003.zip › Software/02_Amplimetrics-V1.2/test_data/Timelapse_Image54.jpg]

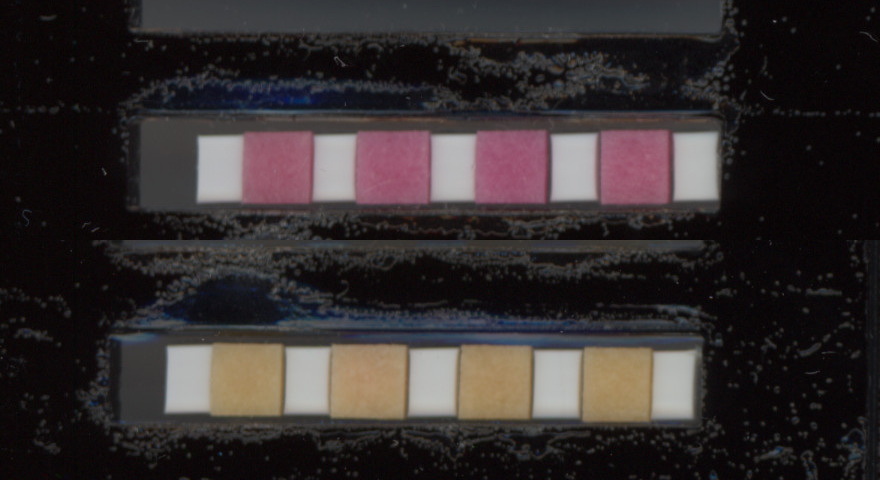

Supplement: S3 File — Amplimetrics™ software as well as ThermiQuant™ AquaStream source codes. (ZIP) [file pone.0348607.s003.zip › Software/02_Amplimetrics-V1.2/test_data/Timelapse_Image55.jpg]

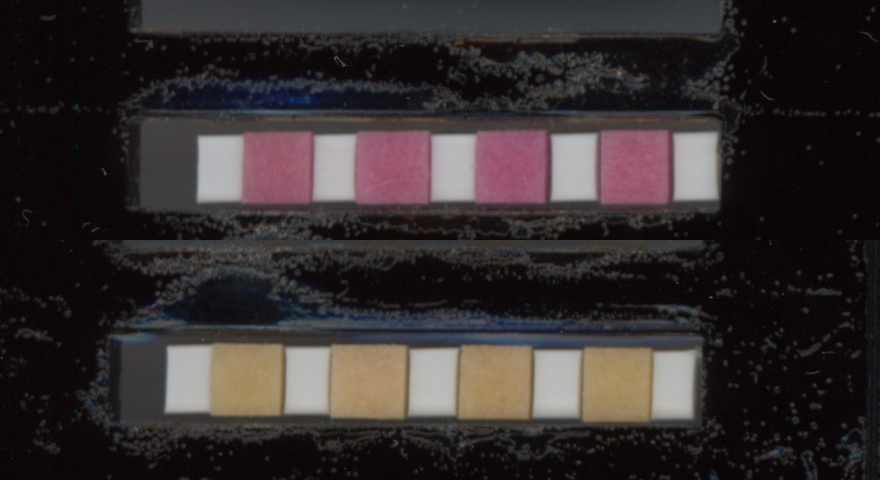

Supplement: S3 File — Amplimetrics™ software as well as ThermiQuant™ AquaStream source codes. (ZIP) [file pone.0348607.s003.zip › Software/02_Amplimetrics-V1.2/test_data/Timelapse_Image56.jpg]

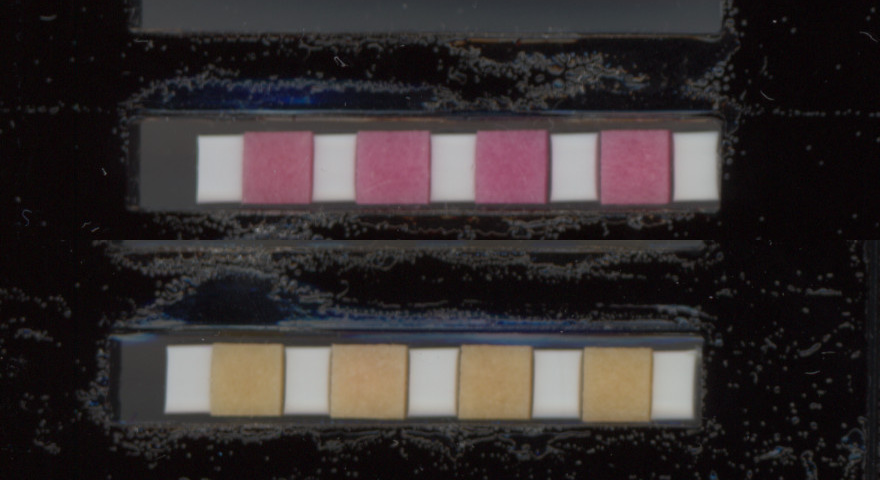

Supplement: S3 File — Amplimetrics™ software as well as ThermiQuant™ AquaStream source codes. (ZIP) [file pone.0348607.s003.zip › Software/02_Amplimetrics-V1.2/test_data/Timelapse_Image57.jpg]

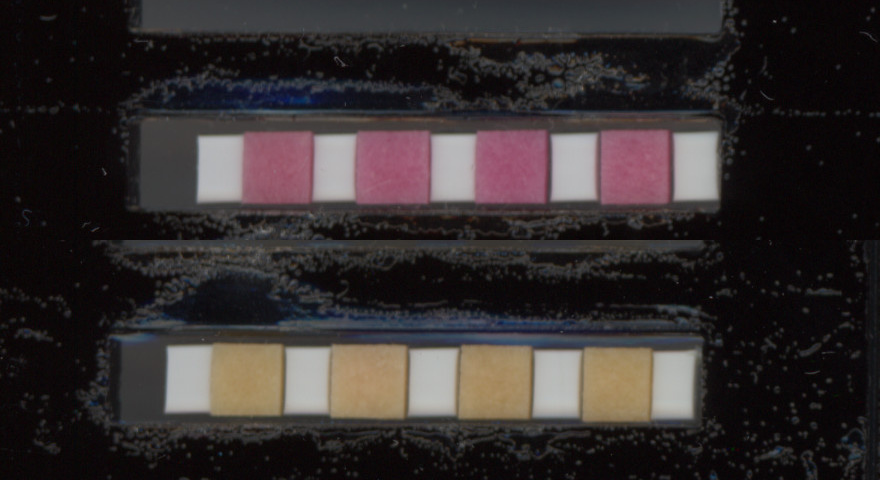

Supplement: S3 File — Amplimetrics™ software as well as ThermiQuant™ AquaStream source codes. (ZIP) [file pone.0348607.s003.zip › Software/02_Amplimetrics-V1.2/test_data/Timelapse_Image58.jpg]

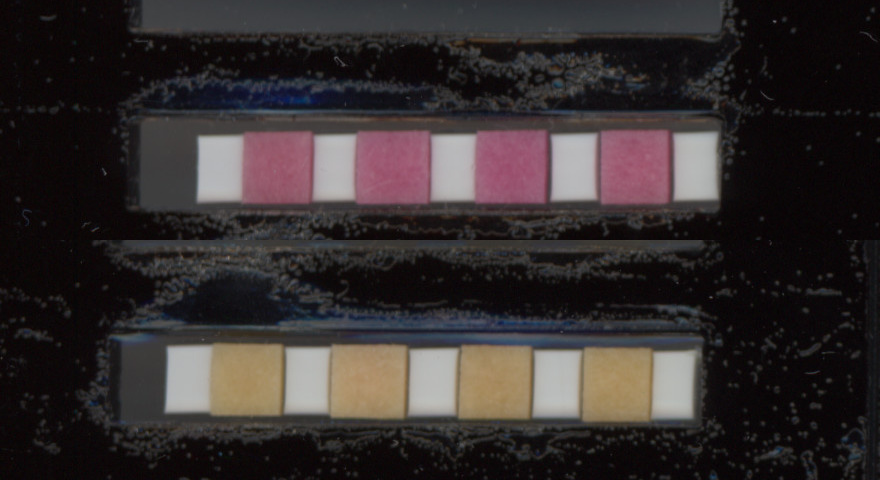

Supplement: S3 File — Amplimetrics™ software as well as ThermiQuant™ AquaStream source codes. (ZIP) [file pone.0348607.s003.zip › Software/02_Amplimetrics-V1.2/test_data/Timelapse_Image59.jpg]

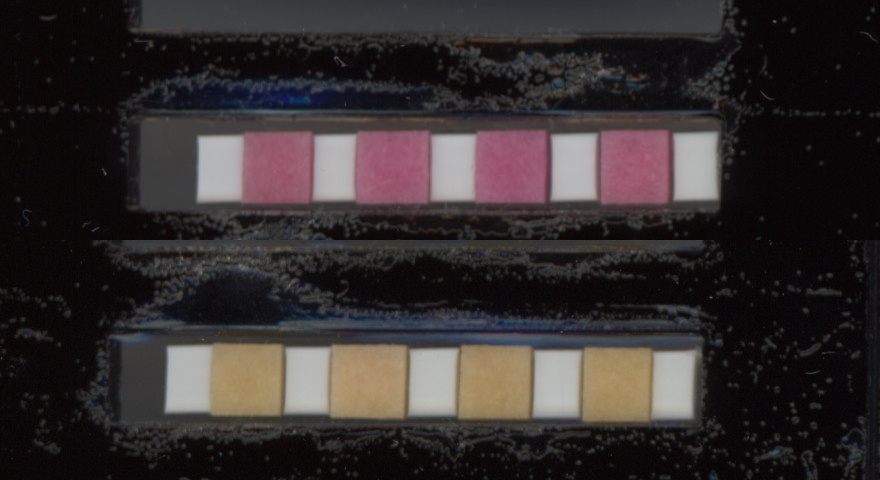

Supplement: S3 File — Amplimetrics™ software as well as ThermiQuant™ AquaStream source codes. (ZIP) [file pone.0348607.s003.zip › Software/02_Amplimetrics-V1.2/test_data/Timelapse_Image60.jpg]

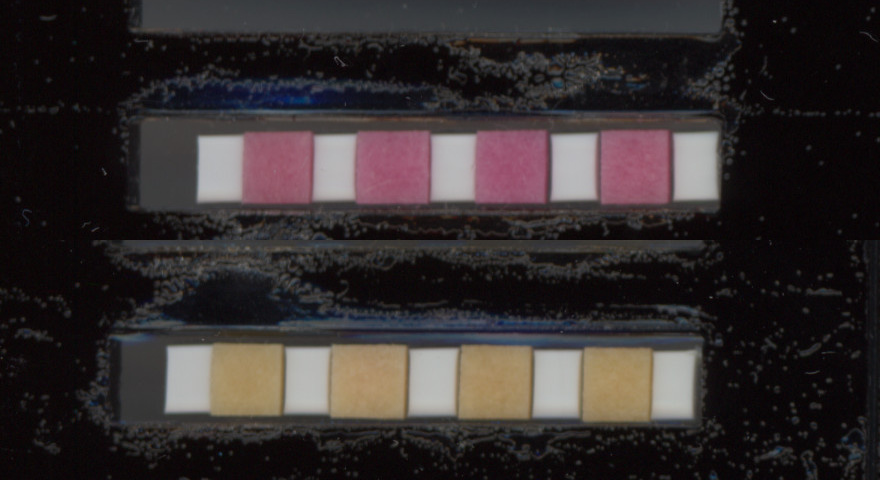

Supplement: S3 File — Amplimetrics™ software as well as ThermiQuant™ AquaStream source codes. (ZIP) [file pone.0348607.s003.zip › Software/02_Amplimetrics-V1.2/test_data/Timelapse_Image61.jpg]

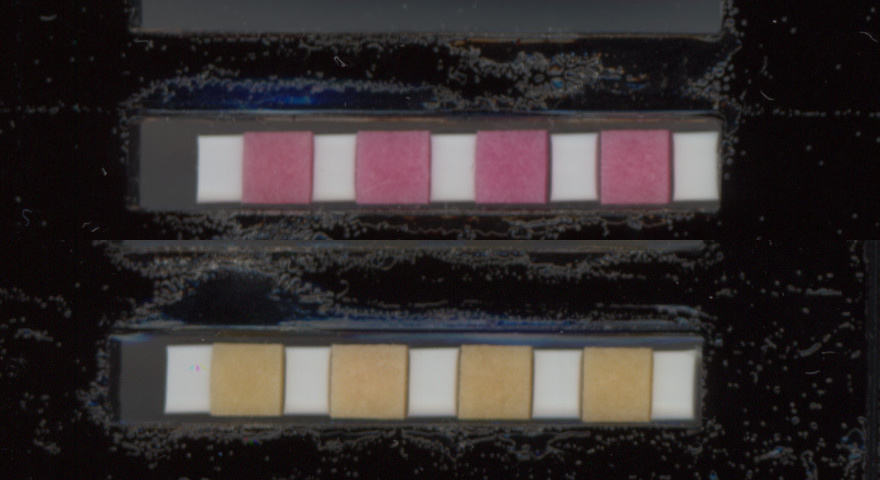

Supplement: S3 File — Amplimetrics™ software as well as ThermiQuant™ AquaStream source codes. (ZIP) [file pone.0348607.s003.zip › Software/02_Amplimetrics-V1.2/test_data/Timelapse_Image62.jpg]

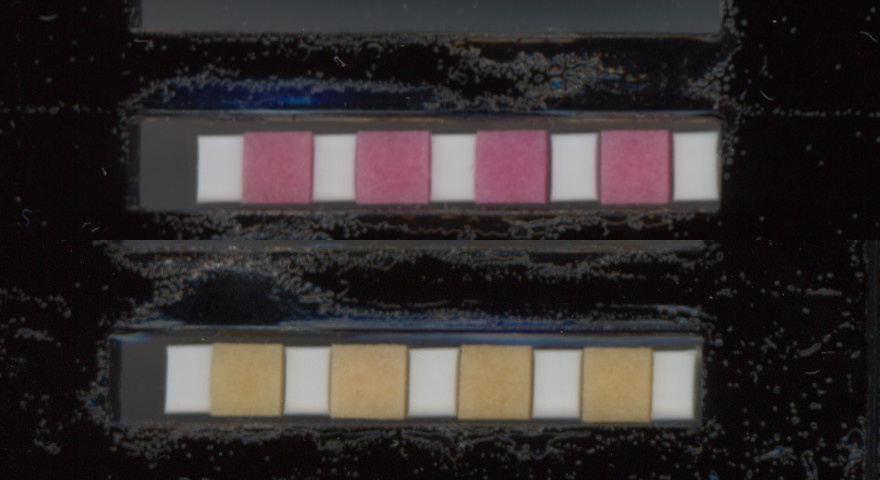

Supplement: S3 File — Amplimetrics™ software as well as ThermiQuant™ AquaStream source codes. (ZIP) [file pone.0348607.s003.zip › Software/02_Amplimetrics-V1.2/test_data/Timelapse_Image63.jpg]

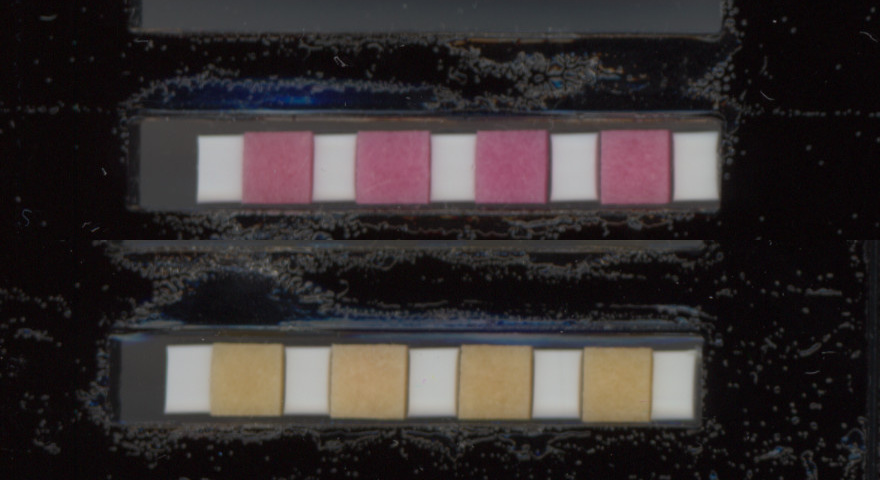

Supplement: S3 File — Amplimetrics™ software as well as ThermiQuant™ AquaStream source codes. (ZIP) [file pone.0348607.s003.zip › Software/02_Amplimetrics-V1.2/test_data/Timelapse_Image64.jpg]

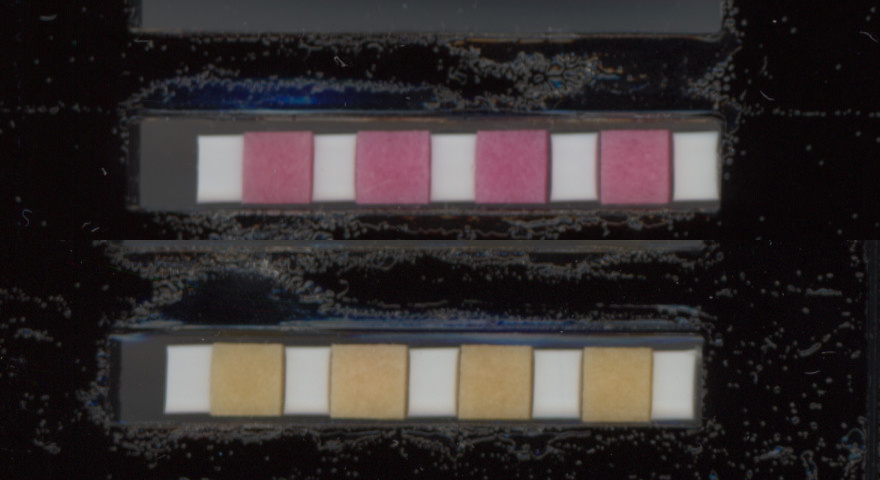

Supplement: S3 File — Amplimetrics™ software as well as ThermiQuant™ AquaStream source codes. (ZIP) [file pone.0348607.s003.zip › Software/02_Amplimetrics-V1.2/test_data/Timelapse_Image65.jpg]

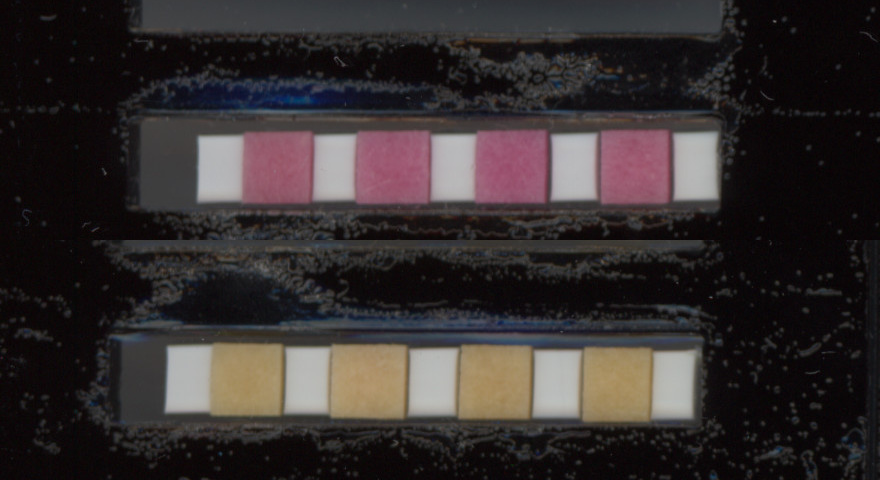

Supplement: S3 File — Amplimetrics™ software as well as ThermiQuant™ AquaStream source codes. (ZIP) [file pone.0348607.s003.zip › Software/02_Amplimetrics-V1.2/test_data/Timelapse_Image66.jpg]

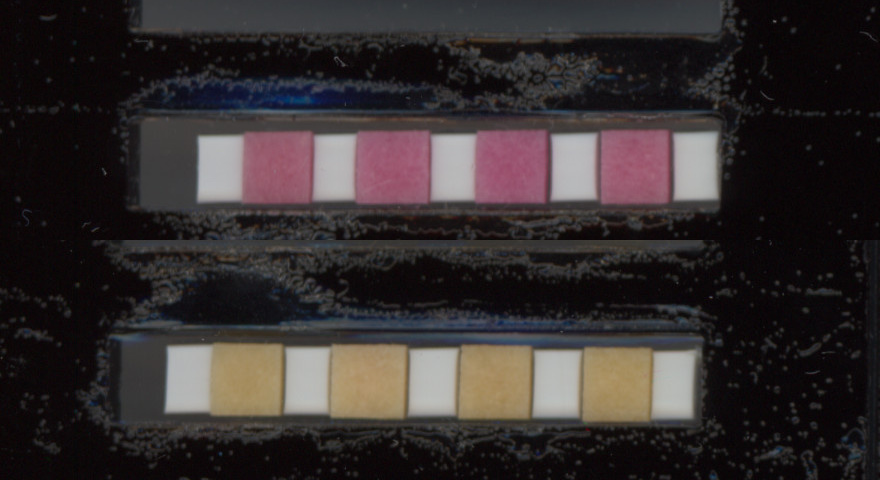

Supplement: S3 File — Amplimetrics™ software as well as ThermiQuant™ AquaStream source codes. (ZIP) [file pone.0348607.s003.zip › Software/02_Amplimetrics-V1.2/test_data/Timelapse_Image67.jpg]

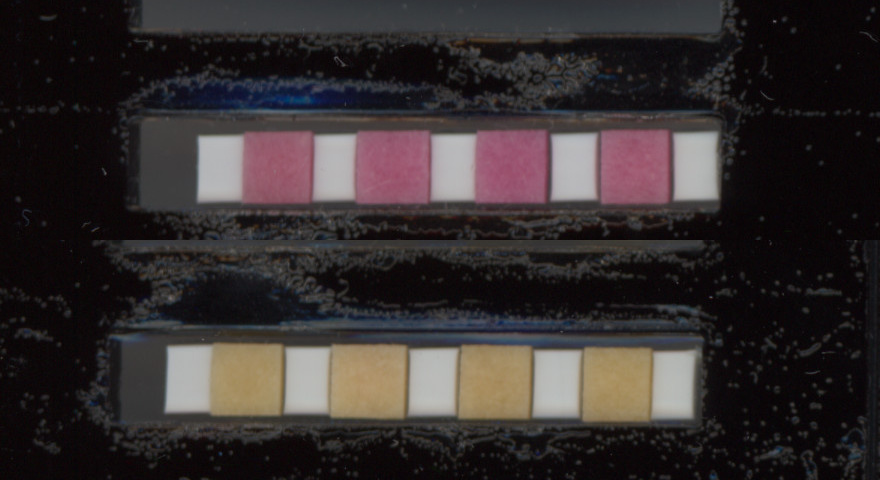

Supplement: S3 File — Amplimetrics™ software as well as ThermiQuant™ AquaStream source codes. (ZIP) [file pone.0348607.s003.zip › Software/02_Amplimetrics-V1.2/test_data/Timelapse_Image68.jpg]

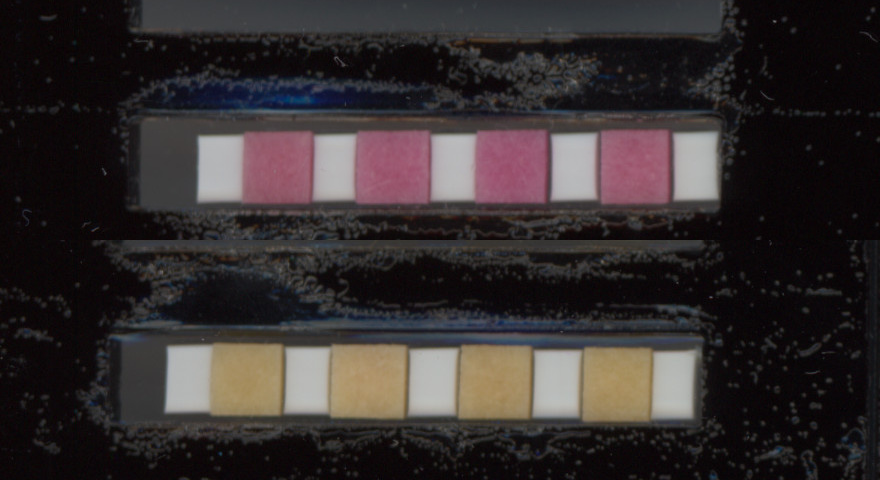

Supplement: S3 File — Amplimetrics™ software as well as ThermiQuant™ AquaStream source codes. (ZIP) [file pone.0348607.s003.zip › Software/02_Amplimetrics-V1.2/test_data/Timelapse_Image69.jpg]

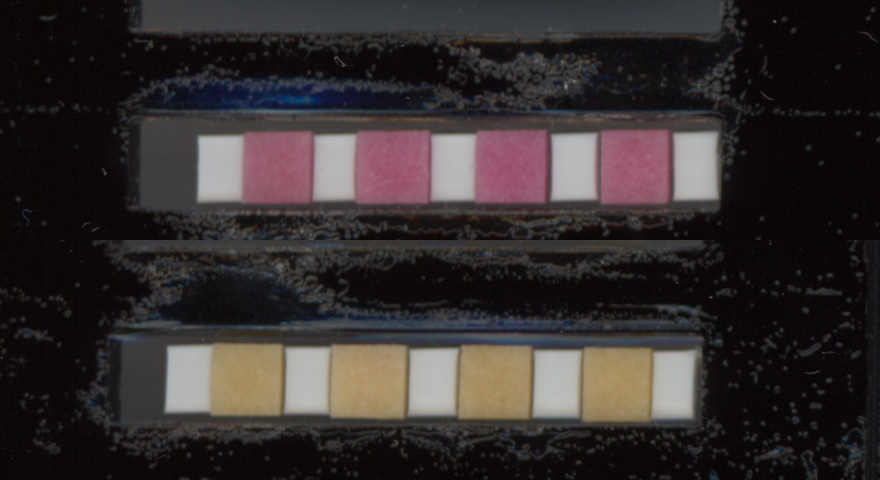

Supplement: S3 File — Amplimetrics™ software as well as ThermiQuant™ AquaStream source codes. (ZIP) [file pone.0348607.s003.zip › Software/02_Amplimetrics-V1.2/test_data/Timelapse_Image70.jpg]

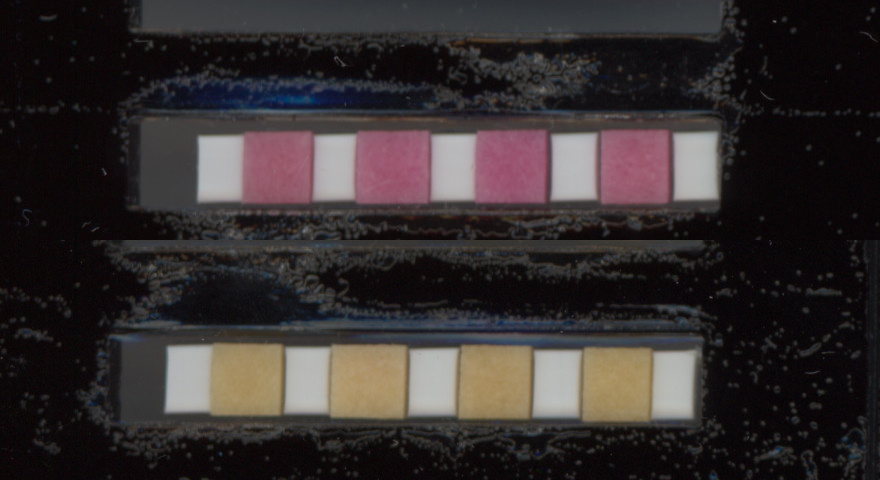

Supplement: S3 File — Amplimetrics™ software as well as ThermiQuant™ AquaStream source codes. (ZIP) [file pone.0348607.s003.zip › Software/02_Amplimetrics-V1.2/test_data/Timelapse_Image71.jpg]

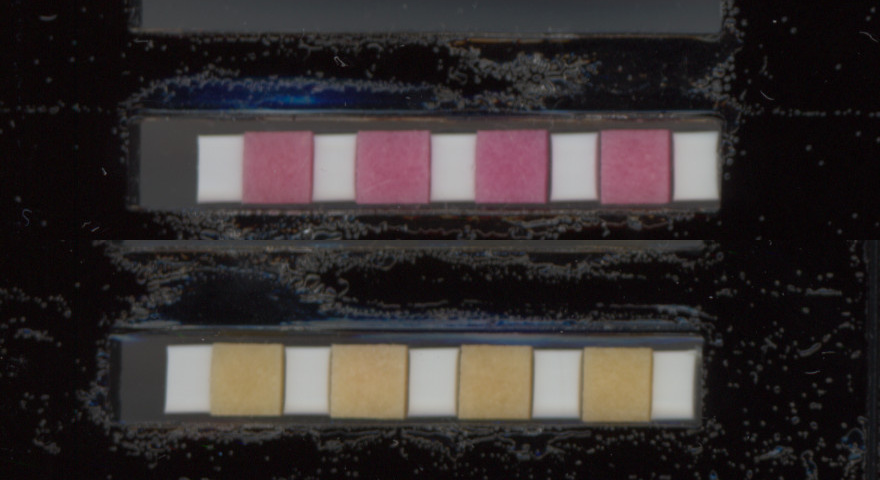

Supplement: S3 File — Amplimetrics™ software as well as ThermiQuant™ AquaStream source codes. (ZIP) [file pone.0348607.s003.zip › Software/02_Amplimetrics-V1.2/test_data/Timelapse_Image72.jpg]

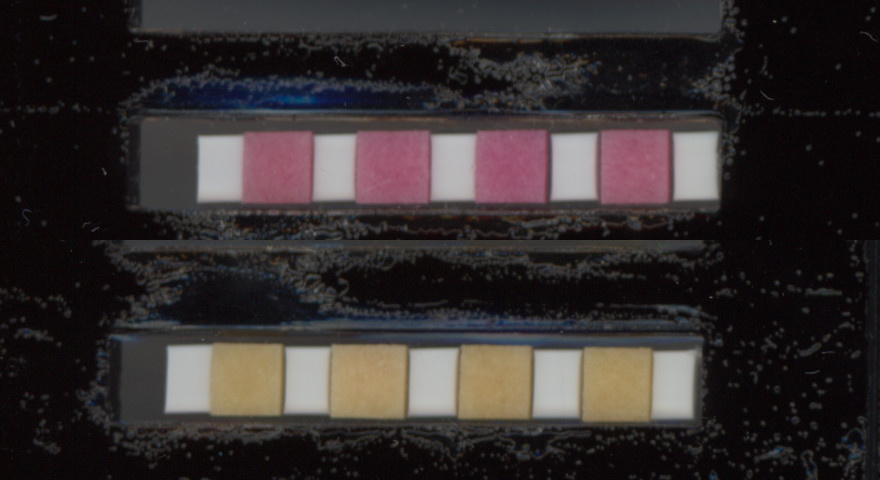

Supplement: S3 File — Amplimetrics™ software as well as ThermiQuant™ AquaStream source codes. (ZIP) [file pone.0348607.s003.zip › Software/02_Amplimetrics-V1.2/test_data/Timelapse_Image73.jpg]

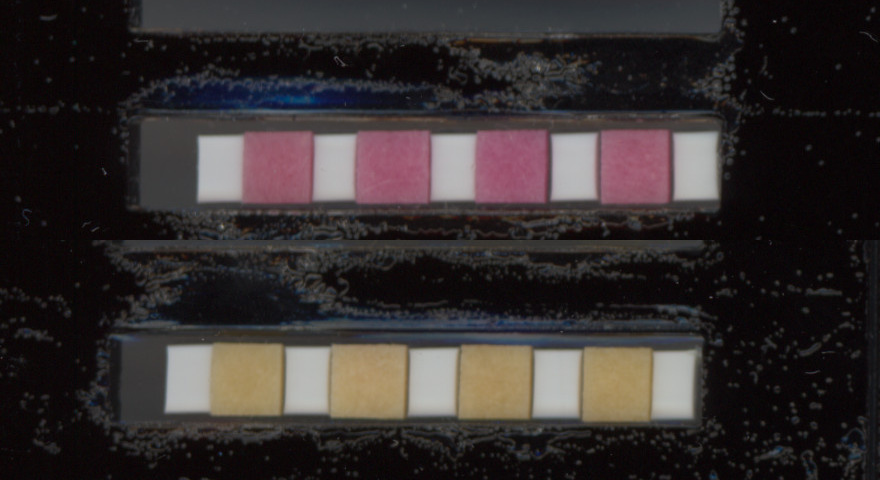

Supplement: S3 File — Amplimetrics™ software as well as ThermiQuant™ AquaStream source codes. (ZIP) [file pone.0348607.s003.zip › Software/02_Amplimetrics-V1.2/test_data/Timelapse_Image74.jpg]

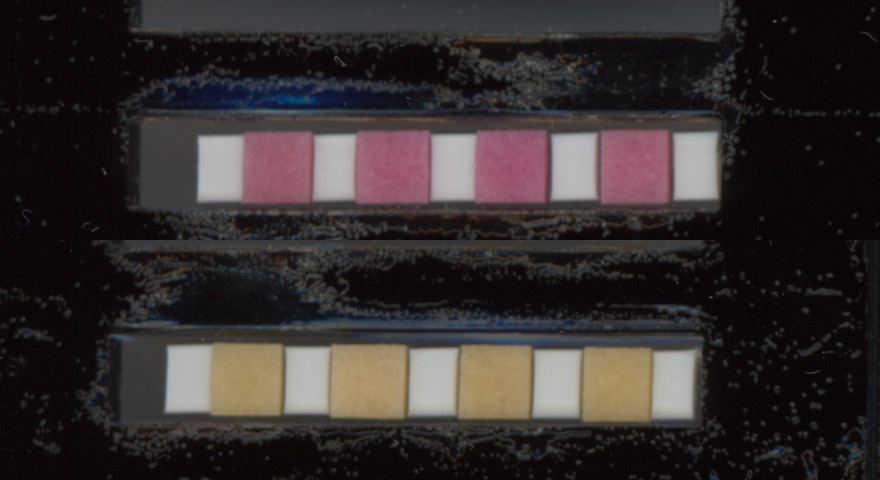

Supplement: S3 File — Amplimetrics™ software as well as ThermiQuant™ AquaStream source codes. (ZIP) [file pone.0348607.s003.zip › Software/02_Amplimetrics-V1.2/test_data/Timelapse_Image75.jpg]

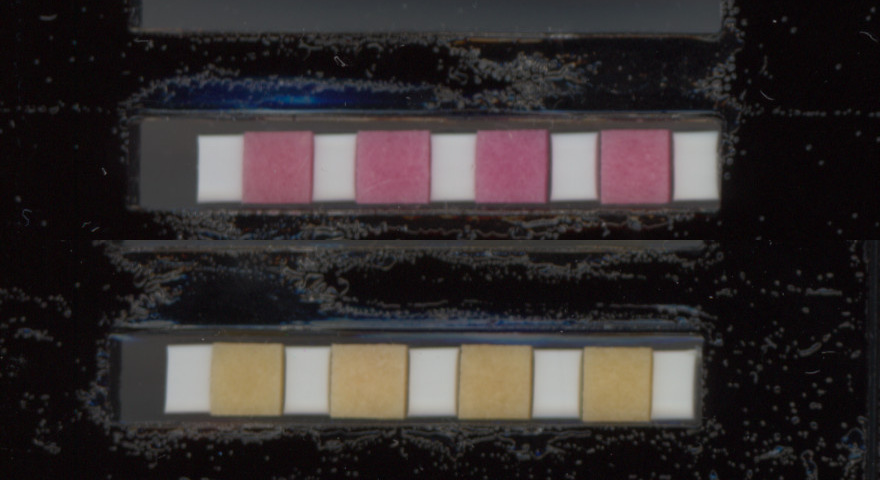

Supplement: S3 File — Amplimetrics™ software as well as ThermiQuant™ AquaStream source codes. (ZIP) [file pone.0348607.s003.zip › Software/02_Amplimetrics-V1.2/test_data/Timelapse_Image76.jpg]

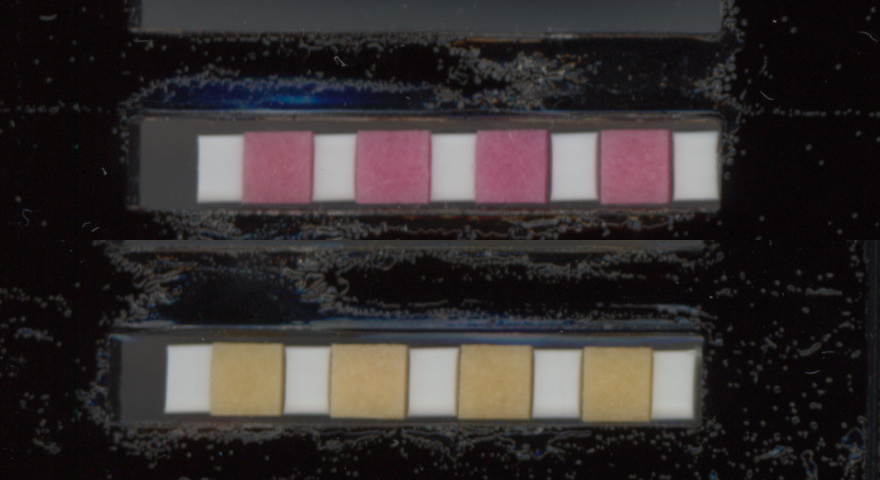

Supplement: S3 File — Amplimetrics™ software as well as ThermiQuant™ AquaStream source codes. (ZIP) [file pone.0348607.s003.zip › Software/02_Amplimetrics-V1.2/test_data/Timelapse_Image77.jpg]

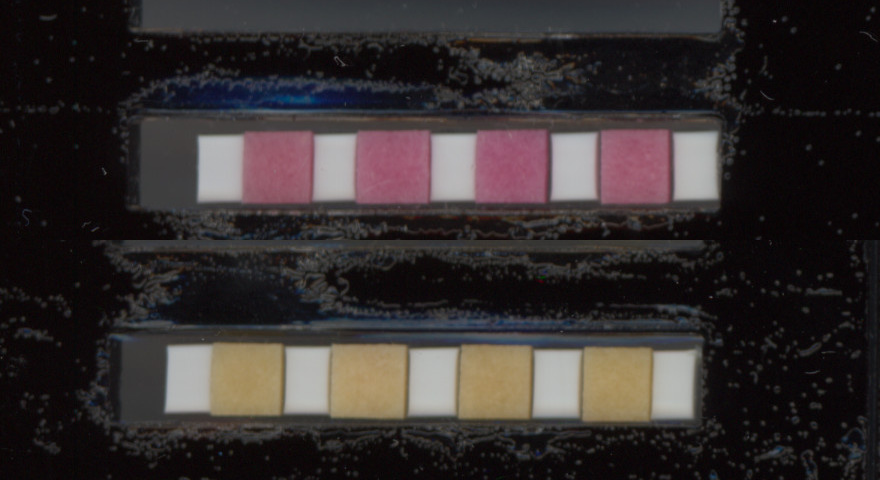

Supplement: S3 File — Amplimetrics™ software as well as ThermiQuant™ AquaStream source codes. (ZIP) [file pone.0348607.s003.zip › Software/02_Amplimetrics-V1.2/test_data/Timelapse_Image78.jpg]

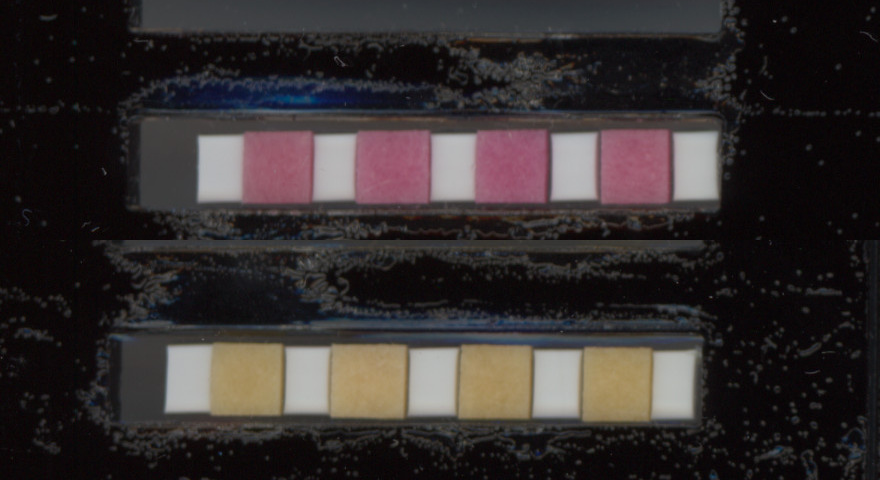

Supplement: S3 File — Amplimetrics™ software as well as ThermiQuant™ AquaStream source codes. (ZIP) [file pone.0348607.s003.zip › Software/02_Amplimetrics-V1.2/test_data/Timelapse_Image79.jpg]

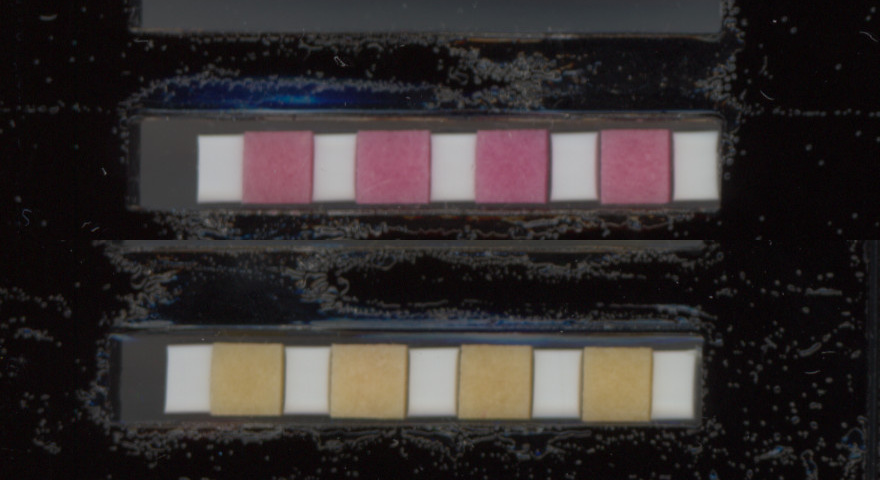

Supplement: S3 File — Amplimetrics™ software as well as ThermiQuant™ AquaStream source codes. (ZIP) [file pone.0348607.s003.zip › Software/02_Amplimetrics-V1.2/test_data/Timelapse_Image80.jpg]

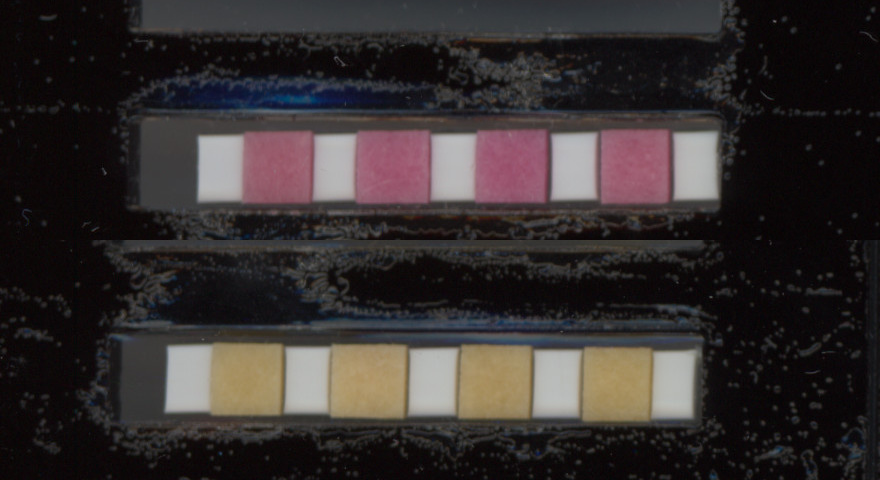

Supplement: S3 File — Amplimetrics™ software as well as ThermiQuant™ AquaStream source codes. (ZIP) [file pone.0348607.s003.zip › Software/02_Amplimetrics-V1.2/test_data/Timelapse_Image81.jpg]

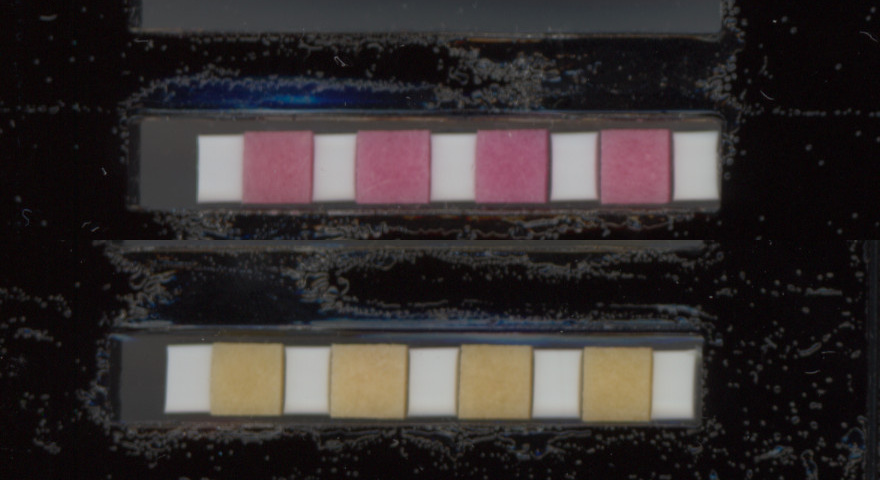

Supplement: S3 File — Amplimetrics™ software as well as ThermiQuant™ AquaStream source codes. (ZIP) [file pone.0348607.s003.zip › Software/02_Amplimetrics-V1.2/test_data/Timelapse_Image82.jpg]

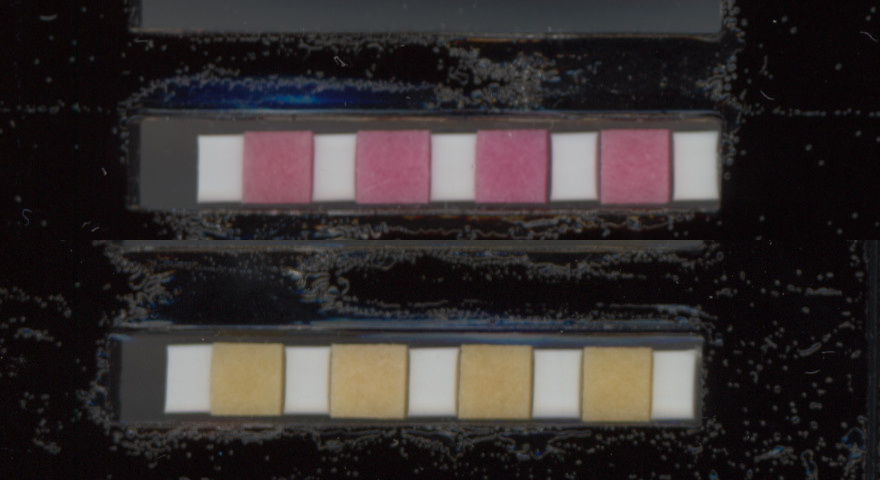

Supplement: S3 File — Amplimetrics™ software as well as ThermiQuant™ AquaStream source codes. (ZIP) [file pone.0348607.s003.zip › Software/02_Amplimetrics-V1.2/test_data/Timelapse_Image83.jpg]

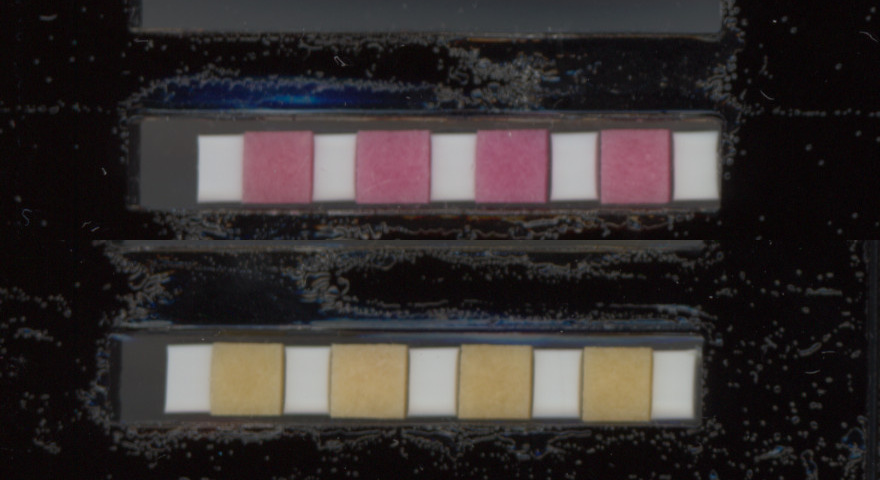

Supplement: S3 File — Amplimetrics™ software as well as ThermiQuant™ AquaStream source codes. (ZIP) [file pone.0348607.s003.zip › Software/02_Amplimetrics-V1.2/test_data/Timelapse_Image84.jpg]

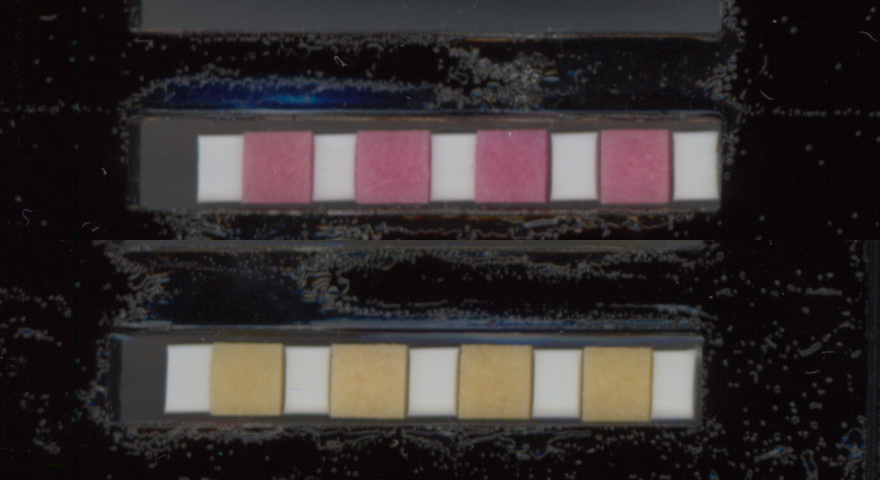

Supplement: S3 File — Amplimetrics™ software as well as ThermiQuant™ AquaStream source codes. (ZIP) [file pone.0348607.s003.zip › Software/02_Amplimetrics-V1.2/test_data/Timelapse_Image85.jpg]

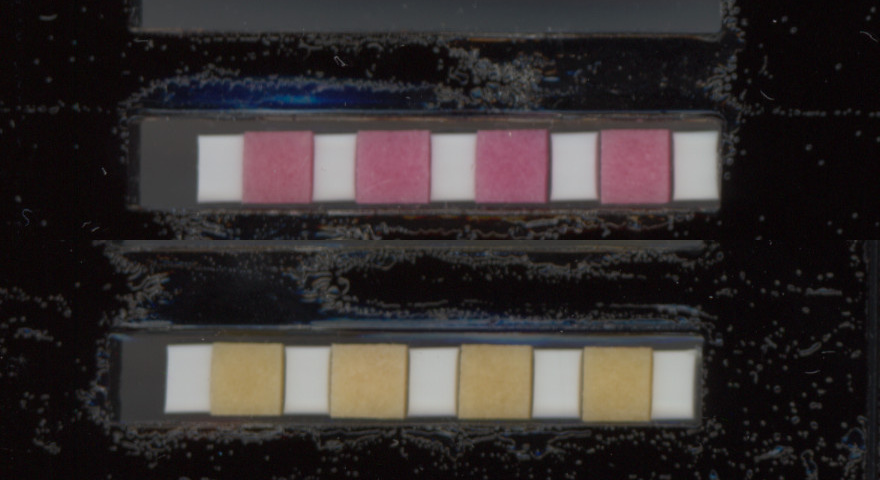

Supplement: S3 File — Amplimetrics™ software as well as ThermiQuant™ AquaStream source codes. (ZIP) [file pone.0348607.s003.zip › Software/02_Amplimetrics-V1.2/test_data/Timelapse_Image86.jpg]

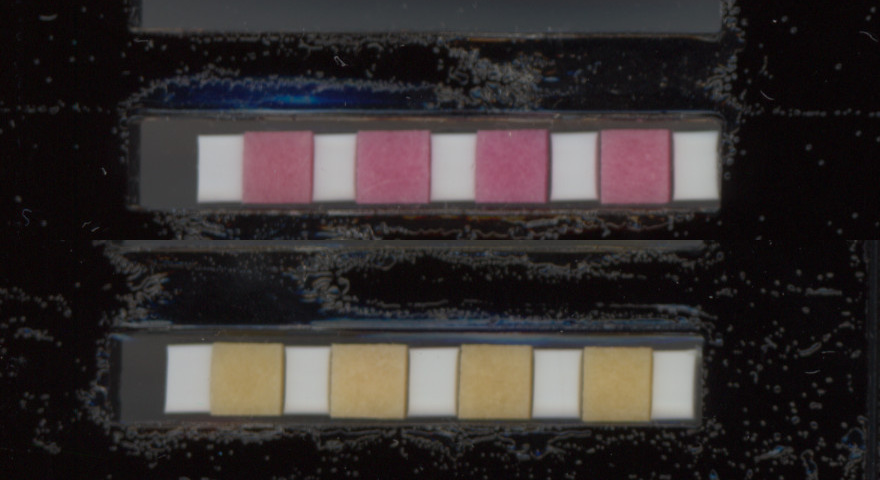

Supplement: S3 File — Amplimetrics™ software as well as ThermiQuant™ AquaStream source codes. (ZIP) [file pone.0348607.s003.zip › Software/02_Amplimetrics-V1.2/test_data/Timelapse_Image87.jpg]

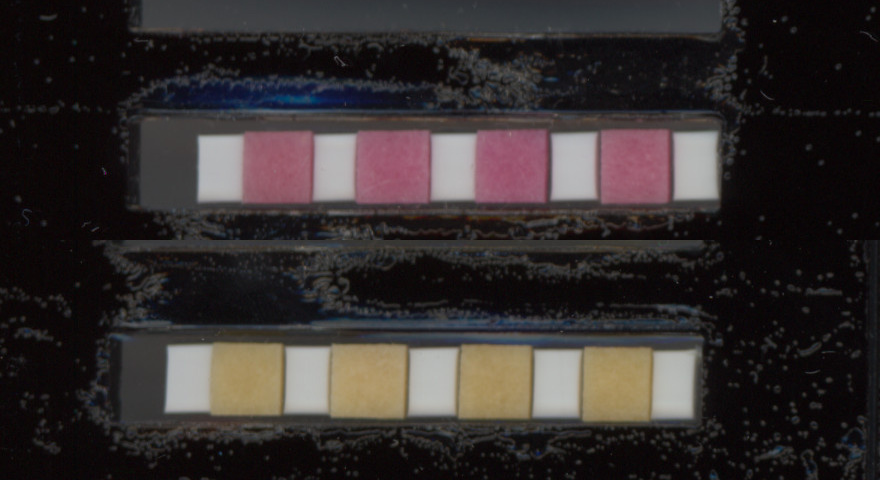

Supplement: S3 File — Amplimetrics™ software as well as ThermiQuant™ AquaStream source codes. (ZIP) [file pone.0348607.s003.zip › Software/02_Amplimetrics-V1.2/test_data/Timelapse_Image88.jpg]

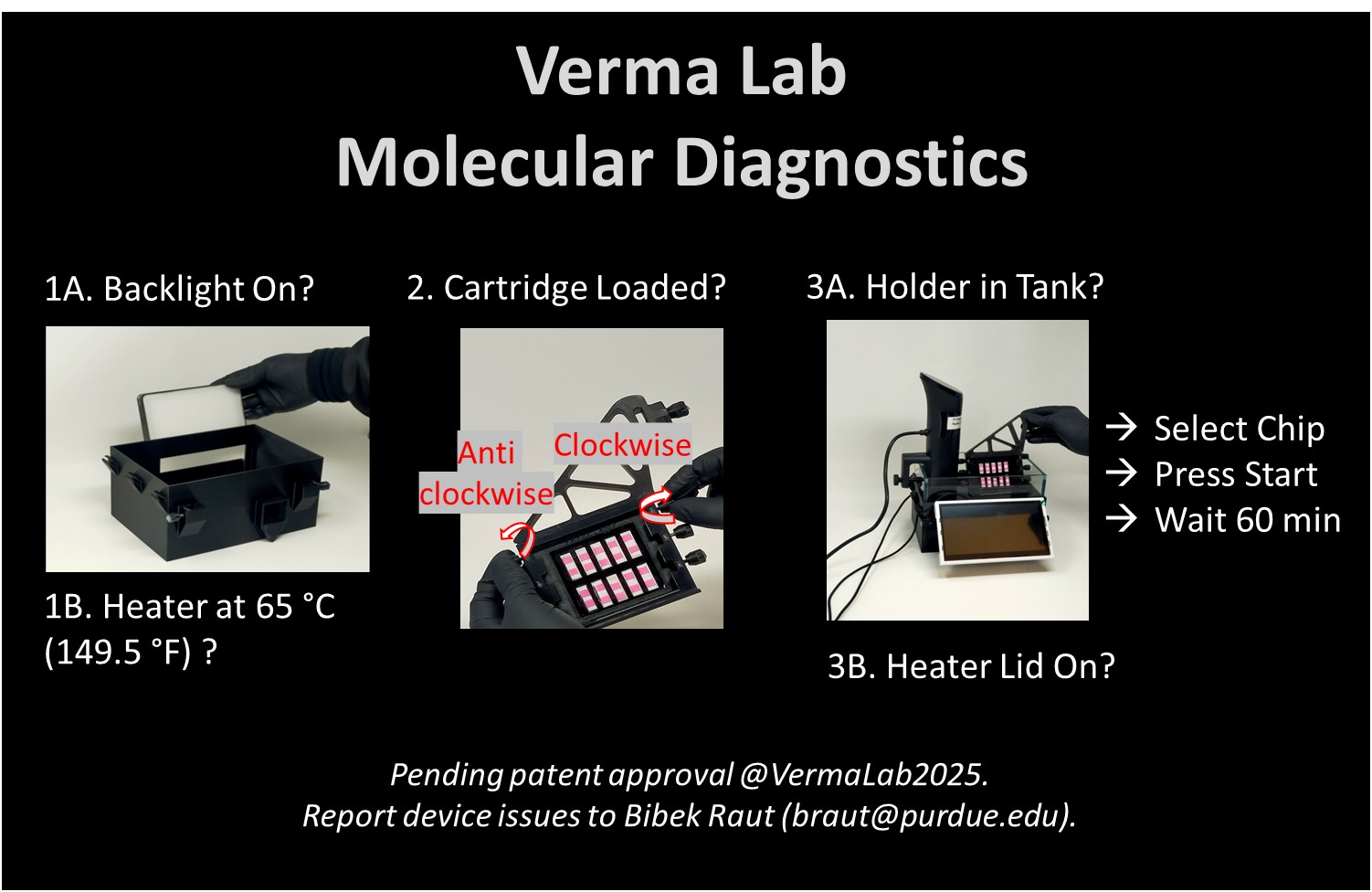

Supplement: S3 File — Amplimetrics™ software as well as ThermiQuant™ AquaStream source codes. (ZIP) [file pone.0348607.s003.zip › Software/03_AquaStream_V1.1/first_image.jpg]
